# Boosting Alcohol Oxidation Electrocatalysis with Multifactorial Engineered Pd_1_/Pt Single-Atom Alloy-BiO_x_ Adatoms Surface

**DOI:** 10.1007/s40820-025-01678-4

**Published:** 2025-03-03

**Authors:** Yujia Liao, Wen Chen, Yutian Ding, Lei Xie, Qi Yang, Qilong Wu, Xianglong Liu, Jinliang Zhu, Renfei Feng, Xian-Zhu Fu, Shuiping Luo, Jing-Li Luo

**Affiliations:** 1 Shenzhen Key Laboratory of Energy Electrocatalytic Materials, Guangdong Provincial Key Laboratory of New Energy Materials Service Safety, College of Materials Science and Engineering, Shenzhen University, Shenzhen, 518055 People’s Republic of China; 2 Department of Chemistry, City University of Hong Kong, Kowloon, 999077 People’s Republic of China; 3 Department of Chemistry, Southern University of Science and Technology (SUSTech), Shenzhen, 518055 People’s Republic of China; 4 School of Resources, Environment and Materials, MOE Key Laboratory of New Processing Technology for Nonferrous Metals and Materials, Guangxi University, Nanning, 530004 People’s Republic of China; 5 Canadian Light Source Inc., 44 Innovation Blvd., Saskatoon, SK S7N 0×4 Canada

**Keywords:** Electrocatalysis, Alcohol oxidation, Single-atom alloy, Intermetallic, Fuel cell

## Abstract

Engineering nanomaterials at single-atomic sites could enable unprecedented catalytic properties for broad applications, yet it remains challenging to do so on the surface of multimetallic nanocrystals. Herein, we present the multifactorial engineering (size, shape, phase, and composition) of the fully ordered PtBi nanoplates at atomic level, achieving a unique catalyst surface where the face-centered cubic (*fcc*) Pt edges are modified by the isolated Pd atoms and BiO_x_ adatoms. This Pd_1_/Pt-BiO_x_ electrocatalyst exhibits an ultrahigh mass activity of 16.01 A mg^−1^_Pt+Pd_ toward ethanol oxidation in alkaline electrolyte and enables a direct ethanol fuel cell of peak power density of 56.7 mW cm^−2^. The surrounding BiO_x_ adatoms are critical for mitigating CO-poisoning on the Pt surface, and the Pd_1_/Pt single-atom alloy further facilitates the electrooxidation of CH_3_CH_2_OH. This work offers new insights into the rational design and construction of sophisticated catalyst surface at single-atomic sites for highly efficient electrocatalysis.

**Supplementary Information:**

The online version contains supplementary material available at 10.1007/s40820-025-01678-4.

## Highlights

- A unique catalyst surface where ultrathin Pt edges are modified by the isolated Pd atoms and BiO_x_ adatoms is rational designed and achieved.
- The Pd_1_/Pt-BiO_x_ electrocatalyst exhibits an ultrahigh mass activity of 16.01 A mg^−1^_Pt+Pd_ toward ethanol oxidation and enables a direct ethanol fuel cell of peak power density of 56.7 mW cm^−2^.
- The surrounding BiO_x_ adatoms are critical for mitigating CO-poisoning on Pt surface, and the Pd_1_/Pt single-atom alloy further facilitates the electrooxidation of CH_3_CH_2_OH.

**Supplementary Information:**

The online version contains supplementary material available at 10.1007/s40820-025-01678-4.

## Graphical abstract

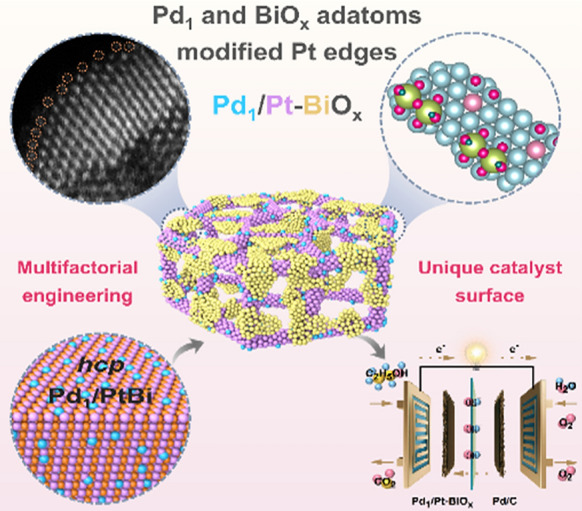

## Introduction

Direct alcohol fuel cells (DAFCs) represent attractive alternatives to hydrogen fuel cells for portable applications, due to their adoption of liquid alcohols (methanol, ethanol, etc.) as fuels, which are more convenient in production, storage, transportation, and refueling. However, the electrooxidations of alcohols are kinetically sluggish processes which suffer from the issues of high overpotentials, intermediates poisoning, and a large dosage of noble metals [1]. Pt is one of the most active monometallic electrocatalysts for alcohol oxidation reactions (AORs), yet commercial Pt catalysts still lack satisfactory catalytic performance for practical DAFCs [2–4]. Engineering multimetallic nanocrystals is one of the most effective strategies to boost AORs electrocatalysis [5–7]. As a notable example, the ternary Pt/Rh/SnO_2_ electrocatalyst combines the specific properties of its components, consequently enables the alcohol dehydrogenation, C–C bond breaking, and oxidation of dissociated CO, thus facilitating the oxidation of ethanol [8]. However, it remains challenging to control, at atomic-level, the multimetallic ensembles on the surface of catalysts, in order to maximize the multifunctional effects and minimize the usage of noble metals [9–23].

Great progress has been made in developing multimetallic electrocatalysts by tuning the size, shape, phase, composition, and surface of multimetallic nanocatalysts. In this regard, single-atom alloy emerges as a high-performance and low-cost electrocatalyst for broad applications, since it combines the multifunctional effects introduced by alloying with the ultrahigh atom utilization of single-atom catalysis [24]. On the other hand, it is well established that some oxyphilic adatoms (Bi, Pb, Sn, etc.) enable the formation of adsorbed hydroxyl species (–OH_ad_) at lower potentials, which can mitigate the poisoning of CO species on Pt surface [25–31]. Different from the regulation of single structural parameters, i.e., size, shape, phase, composition, or surface, the multifactorial engineering strategy holds promise in developing unprecedented electrocatalysts via constructing well-defined multimetallic ensembles. However, the multifactorial engineering of structural parameters at atomic-level in a nanocrystal still remains as a challenging task [32–45].

In this work, starting from the hexagonal close packed (*hcp*) PtBi intermetallic nanoplates and via a rational design, we constructed a novel catalyst surface, namely, Pt edges modified by atomically dispersed noble metal atoms (*M* = Pd, Rh, or Ir) and BiO_x_ adatoms (M/Pt-BiO_x_). Intriguingly, the size, shape, phase, composition, and surface of the fully ordered PtBi nanoplates are transformed all-in-one by a facile wet-chemistry method and subsequent annealing treatment. The Pd_1_/Pt-BiO_x_ electrocatalyst thus constructed exhibits a peak mass activity of 16.01 A mg^−1^_Pt+Pd_ toward ethanol oxidation reaction (EOR), while the Rh/Pt-BiO_x_ shows a peak mass activity of 15.31 A mg^−1^_Pt+Rh_ toward methanol oxidation reaction (MOR), both in alkaline electrolytes, these values are 16.4 and 8.3 times higher than those of commercial Pt/C catalysts, respectively. Density functional theory (DFT) calculations suggest that the BiO_x_ adatoms boost the EOR electrocatalysis via mitigating the CO-poisoning on *fcc*-Pt surface, and the dilute Pd atoms on the Pt surface further contribute to their ultrahigh electrocatalytic performance.

## Experimental Section

### Chemicals and Materials

Platinum acetylacetonate (Pt(acac)_2_, 98%), bismuth acetate (Bi(act)_3_, 99.99%), cetyltrimethyl ammonium bromide (CTAB, 99%), octadecene (ODE, 90%), oleylamine (OAM, 70%) and ascorbic acid (AA, 99%) were purchased from Sigma-Aldrich. Palladium acetylacetonate (Pd(acac)_2_, 99%) and iridium chloride hydrate (IrCl_3_·xH_2_O, Ir > 52%) were purchased from Aladdin. Rhodium acetylacetonate (Rh(acac)_3_, 97%) was purchased from Macklin. All chemicals were used as received without further purification. Liquids such as n-butylamine, acetic acid, sulfuric acid, hexane, and ethanol were analytical grade and used as received without further purification. The water used in all experiments was ultrapure (18.2 MΩ cm).

### Syntheses of M/PtBi (M = Pd, Rh, Ir, or Ru) Intermetallic Nanoplates

In a typical synthesis of M/PtBi (*M* = Pd, Rh, or Ir) intermetallic nanoplates, 80 mg AA, 350 mg CTAB, 20.5 mg Pt(acac)_2_, 20.0 mg Bi(act)_3_, 5 mL ODE, and 5 mL OAM were consecutively added into a 40-mL glass vial. The vial was capped, transferred into a heating module with the temperature kept at 80 °C, and heated for 1 h under magnetic stirring. Then, the vial was transferred into another heating module with the temperature kept at 220 °C, and heated for 1 h under magnetic stirring. After being cooled to 160 °C, the vial was opened by removing the cap and 1.4 mg Pd(acac)_2_ was added into the mixture to achieve Pd/PtBi. The vial was capped, transferred into the heating module with the temperature kept at 220 °C, and heated for 1 h. After the vial being cooled at room temperature, Pd/PtBi nanoplates were collected by precipitated and washed with hexane/ethanol solution via centrifugation. Rh/PtBi Ir/PtBi, and Ru/PtBi nanoplates were synthesized by the same procedures, except the adding of 1.8 mg Rh(acac)_3_, 1.4 mg IrCl_3_·xH_2_O, and 1.8 mg Ru(acac)_3_ rather than 1.4 mg Pd(acac)_2_ in the typical synthesis of Pd/PtBi nanoplates.

### Syntheses of M/Pt-BiO_x_

In a typical synthesis, the M/PtBi nanoplates were washed with hexane/n-butylamine/ethanol (6: 0.4: 3.6) solution and further dispersed in butylamine solution and kept at 70 °C for 3 days under magnetic stirring, achieving face-centered cubic (*fcc*) Pt edges and amorphous Bi complex on the surface of M/PtBi nanoplates. Then, the butylamine-treated M/PtBi nanoplates were collected by centrifugation and transferred into acetic acid solution and kept at room temperature for 10 h under magnetic stirring to remove surface amorphous Bi complex mildly. The M/PtBi nanoplates after segregation and etching were collected by centrifugation and redispersed into OAM, and then loaded onto carbon support with high surface area (Vulcan XC72) by sonication for 1 h. The products were then collected by centrifugation and washed three times with hexane/n-butylamine/ethanol mixture (6: 0.4: 3.6) and dried in vacuum at 60 °C for 1 h. The pulverized samples were annealed at 200 °C in air for 1 h to achieve the final product M/Pt-BiO_x_. The as-prepared M/Pt-BiO_x_ can be used as the catalysts for alcohol oxidation reaction (AOR) tests directly.

### Materials Characterizations

The low-magnification transmission electron microscopy (TEM) images were obtained by Hitachi HT7700. High-angle annular dark-field scanning transmission electron microscopy (HAADF-STEM) images and energy-dispersive X-ray spectroscopy (EDX) maps were collected by a FEI Titan Cubed Themis G20 equipped with double corrected spherical aberration (300 kV). Some HAADF-STEM images and EDX maps were collected by the FEI Talos F200X TEM (200 kV). The samples were prepared by dropping hexane dispersion of samples onto 300-mesh Cu grids and immediately evaporating the solvent. X-ray diffraction (XRD) measurements were performed on Rigaku SmartLab Diffraction Workstation using Cu-Kα radiation. The elementary composition and Pt loading of catalysts were determined by ICP-MS (Agilent 7700x). X-ray photoelectron spectrum (XPS) was performed on a PHI 5000 Versaprobe III XPS spectrometer with Al Kα as the excitation source. Ar ion sputtering XPS in-depth analysis was obtained by first sputter the elements of a certain thickness off the surface, and then use XPS to analyze the element content of the fresh surface after stripping, so as to obtain the distribution of elements along the depth of the sample.

### In Situ FTIR Measurement

Before the chemical deposition of Au, the Si crystal was first immersed in aqua regia solution to remove the previous film, then polished with 0.05 μm Al_2_O_3_ powder until the surface became hydrophobic. Then, the prism was successively sonicated in deionized (DI) water, acetone, and DI water for 5 min each to clean the surface. It should be noted that the surface should still be totally hydrophobic after sonication. Otherwise, repolishing should be conducted. Next, the cleaned crystal was immersed in a 40% NH_4_F bath for 2 min to form a hydride-terminated surface. Then, the crystal was immersed in the Au seeding solution containing 3 mL Au plating solution and 200 μL 2% HF, under 55 °C for 2–3 min. Finally, the crystal was rinsed with DI water. The ohmic resistance along the Au film diagonal was around 10 Ω. The Au-coated prism was then assembled into a custom-made spectro-electrochemical cell as the working electrode, an Ag/AgCl reference electrode and a graphite rod counter electrode. The Au films were activated in 0.5 M H_2_SO_4_ via cyclic voltammetry for 3 cycles between 0 and 1.45 V vs. Ag/AgCl with a scan rate of 100 mV s^−1^ in order to improve the signal.

Typically, 1 mg of the catalysts was redispersed in a mixture of 0.5 mL of DI water, 0.5 mL of isopropanol, and 20 μL of 5% Nafion under ultrasonication for 1 h to produce ink solutions. The suspension was then placed onto Au films and then used as the working electrode. The Hg/HgO calibrated by a reversible hydrogen electrode (RHE) were used as the reference in the alkaline environment. A graphite rod served as the counter electrode. ATR-SEIRA spectra were recorded using a Nicolet 470 FTIR spectrometer equipped with an MCT detector and a custom-made ATR accessory.

### Electrochemical Study

1.0 mg of the dry catalysts were dispersed in a mixed solvent containing 498 µL of ultrapure water, 498 of µL isopropanol, and 4 μL of Nafion (5 wt%)/ethanol (D520, DuPont) by sonication for 1 h, obtaining a homogeneous catalyst ink. All electrochemical measurements were performed in a three-compartment electrochemical cell with PINE instrument bipotentiostat and CHI 760E electrochemical workstation (Shanghai Chenhua Co., China) at room temperature. The catalyst ink (5 μL, ~ 1 ug of Pt) loaded on glassy carbon (5 mm in diameter) was used as working electrode, a carbon rod was used as a counter electrode, and a saturated calomel electrode (SCE) with salt bridge was used as a reference electrode. All potentials are referred to SCE.

The cyclic voltammetry (CV) curves were obtained at a scan rate of 50 mV s^−1^ in Ar-saturated 1.0 M KOH solution. The methanol and ethanol electrooxidation tests were carried out in Ar-saturated 1.0 M KOH + 1.0 M CH_3_OH and 1.0 M KOH + 1.0 M CH_3_CH_2_OH solutions, respectively. The chronoamperometry (CA) measurements were performed at − 0.4 V vs. SCE. For CO-stripping tests, the working electrode was held at -0.88 V vs. SCE for 15 min under a flow of CO in Ar-saturated 1.0 M KOH. The remaining CO in the electrolyte was thoroughly purged with Ar for 15 min, and then the CV curves were obtained at a scan rate of 50 mV s^−1^.

### DEFC Performance Test

The Pt loading of anode catalyst was 1.24 mg cm^−2^ for the carbon-supported Pd_1_/Pt-BiO_x_ and 1.67 mg cm^−2^ for Pd/C (60 wt%), respectively. To prepare anode catalyst ink, Pd_1_/Pt-BiO_x_/C was added in water (942 μL), isopropanol (1200 μL), and Nafion solution (51.6 μL, 5 wt%), and then sonicated for 60 min. The anode catalyst was coated on gas diffusion layer, which was cut into 1.0 cm × 1.0 cm (1.0 cm^2^) and then kept in 1.0 M KOH for one night. The Pd_1_/Pt-BiO_x_/C-coated gas diffusion layer was activated in Ar-saturated 1.0 M KOH + 1.0 M CH_3_CH_2_OH by CV sweeping at a scan rate of 50 mV s^−1^ for 150 cycles. For the cathode catalyst ink, Pd/C (60 wt%) were dispersed in water (942 μL), isopropanol (1200 μL), and Nafion solution (38.4 μL, 5 wt%), and then sonicated for 60 min. The cathode catalyst was coated on a gas diffusion layer, which was cut into 1.0 cm × 1.0 cm. In order to exchange H^+^ in Nafion, the gas diffusion layer was kept in 1.0 M KOH for one night. The PBI membranes (35 μm) were treated sequentially in 6.0 M KOH solution for 3 h at 60 °C, for 6 h at 40 °C, finally kept in 6.0 M KOH solution for one night. The MEA was fabricated by cold pressing at 2.0 MPa for 10 min. 4.0 M ethanol in 6.0 M KOH was fed to the anode inlet of the MEA at the flow rate of 5 mL min^−1^, and cathodic O_2_ were provided at 200 mL min^−1^. The cell temperature was kept at 60 °C. After keeping the MEA under these conditions for about 0.5 h, a constant open-circuit potential was obtained and then followed by the polarization performance test.

### DFT Calculations

Ab initio calculations were performed with the periodic DFT code Vienna ab initio simulation package (VASP). The exchange and correlation energy were calculated within the generalized gradient approximation (GGA) using the Perdew −Burke−Ernzerhof (PBE) functional. To include van der Waals forces, we added the D3 correction as implemented by Grimme et al. The electron-core interaction was described with the projector augmented wave (PAW) method. The electronic wave functions were expanded using a plane wave basis set with an energy cutoff of 400 eV. All structures were relaxed until the residual forces on the free atoms were smaller than 0.02 eV Å^−1^. The Pt (111) surface were represented using a (4 × 4) supercell consisting of four layers, where the bottom two layers were kept fixed during the relaxations. In all of the cases, 15 Å of a vacuum was added in the z-direction. Geometry optimizations were carried out using Monkhorst–Pack k-point meshes of 3 × 3 × 1. And denser k-point meshes of 6 × 6 × 1 were used for electronic structure analysis. The transition state of C–C cleave was determined by using the improved dimer method (IDM). The electronic structure analysis was assistant by VASPKIT package. Meanwhile, atomic simulation environment (ASE) and VESTA software were used for model building. The reaction Gibbs free energy changes (ΔG) for each of the elementary steps were calculated by the following equation using the computational hydrogen electrode (CHE) model:

1 $$\Delta G = \Delta E + \Delta ZPE - T\Delta S + \Delta G_{U}$$

where ΔE is obtained directly from DFT calculations, ΔZPE is the change of zero-point energies, T is the temperature of 298.15 K, and ΔS is the change in entropy of products and reactants. $\Delta G_{U} = - eU$ is the contribution of electrode potential to ΔG.

## Results and Discussion

**Fig. 1:**
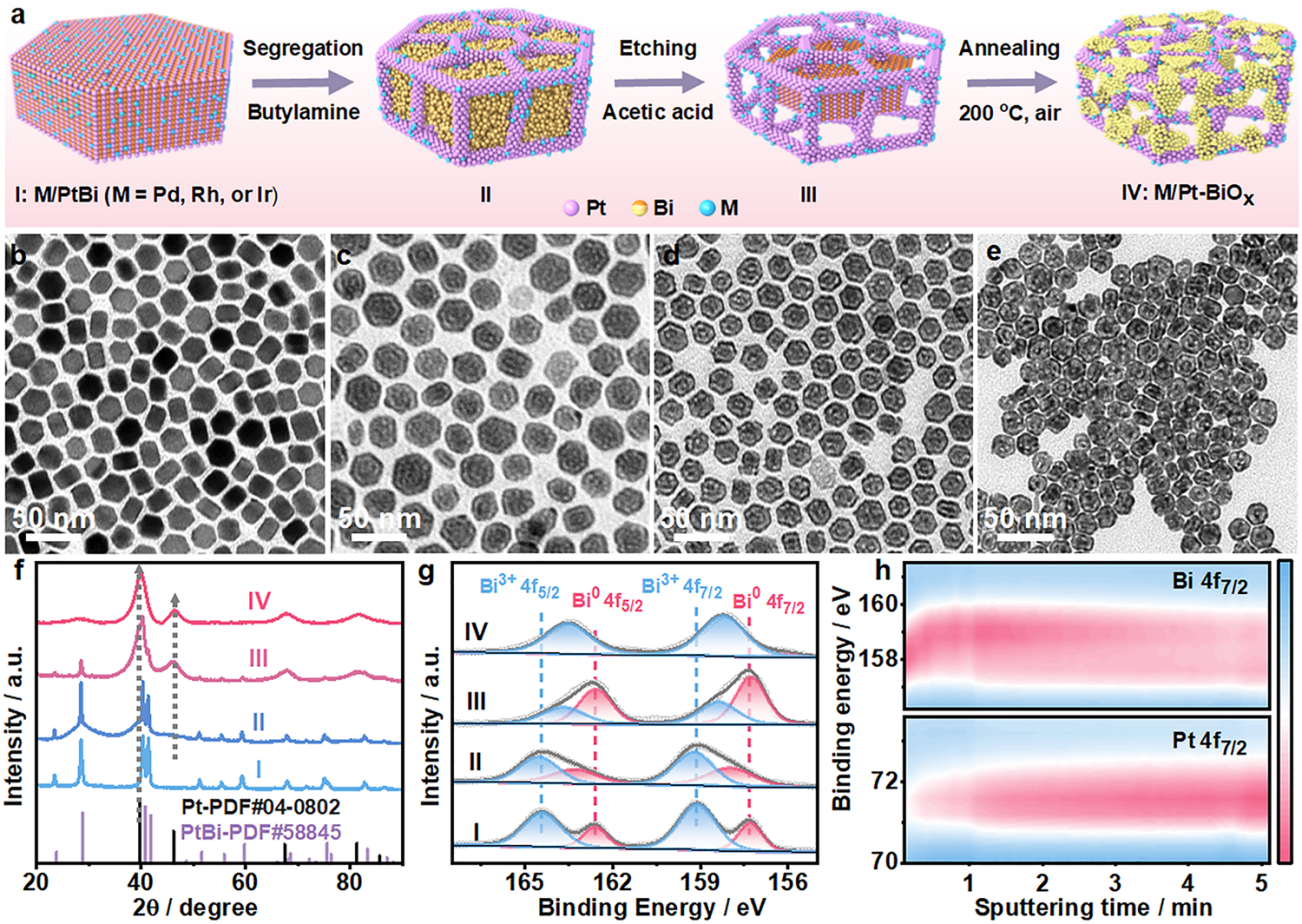
**a** Schematic illustration and **b–e** corresponding TEM images of the preparation procedure of M/Pt-BiO_x_ (*M* = Pd, Rh, or Ir). I, II, III, and IV represent the initial M/PtBi nanoplates, M/PtBi after treatment with butylamine, M/PtBi after treatment with butylamine and acetic acid, and the final M/Pt-BiO_x_ with M/Pt dilute alloy-BiO_x_ adatoms surface, respectively. **f** XRD patterns of I, II, III, and IV. **g** Bi 4*f*_7/2_ XPS spectra of I, II, III, and IV. All of the spectra were calibrated by C 1*s* peak located at 284.8 eV. **h** XPS in-depth analyses of the Pd_1_/Pt-BiO_x_ nanoplates with different sputtering times

Figure 1a illustrates the preparation of M/Pt-BiO_x_ (*M* = Pd, Rh, or Ir) nanoframes based on *hcp* PtBi intermetallic nanoplates, and the experimental details are provided in the Supporting Information. Firstly, Pd, Rh, or Ir was atomically dispersed on PtBi intermetallic nanoplates (I: M/PtBi) by galvanic replacement between isolated Bi atoms and noble metal precursors according to our recent work [46]. As a result, uniform M/PtBi nanoplates (I, Figs. 1b and S1) were fabricated, which show the same crystalline peaks with PtBi intermetallic (JCPDS-PDF#58,845) due to the trace amount of M introduced (Fig. 1f). The hexagonal M/PtBi intermetallic nanoplates have an average diameter of ~ 15.3 nm (Fig. S2). Then, the M/PtBi nanoplates were heated in butylamine solution (pH = 10.7) at 70 °C to induce the rearrangement of Pt and Bi atoms, resulting in the formation of ultrathin *fcc*-Pt edges and amorphous bismuth hydroxide species on the surface [30, 47]. The differences in contrast are obviously observed in the transmission electron microscopy (TEM) image of M/PtBi nanoplates after the treatment with butylamine (II, Figs. 1c and S3a, b), and the additional *fcc*-Pt diffraction peaks appear (Figs. 1f and S3c). The high-resolution TEM image in Fig. S3d shows the typical lattice spacing of 2.30 Å on the edges, which could be indexed as the (111) plane of *fcc*-Pt. In addition, no characteristic peaks of Bi or Bi complex could be observed in the XRD pattern, while the content of Bi is well-maintained (46.9%). As shown in Figs. 1g and S4, the Bi 4*f* peaks of the butylamine-treated nanoplates shift to higher binding energy, and the M–OH peak intensity in the O 1*s* spectrum increases significantly. These results demonstrate the formation of amorphous bismuth hydroxide on the surface [30, 47–49]. Pt 4*f* XPS spectra (Fig. S5a, b) indicate that Pt maintains the dominant metallic state after butylamine treatment and the corresponding binding energy shifted slightly, showing that Pt was less affected compared with Bi during the reaction.

The amorphous bismuth hydroxide and Bi atoms on the surface were easily removed by acetic acid at room temperature, hollowing out the hexagonal nanoplates (III, Figs. 1d and S6). As determined by inductively coupled plasma mass spectrometry (ICP-MS), the atomic content of Bi decreases to ~ 25% (Pt/Bi/Ir = 72.3/25.2/2.5, Pt/Bi/Pd = 70.6/25.4/4.0, Pt/Bi/Rh = 77.9/20.2/1.9) (Table S1), which indicates that the Bi atoms in the top 3 to 4 layers (Fig. S7) of PtBi unit cells are removed from the PtBi intermetallic nanoplates [46]. For M/PtBi nanoplates treated directly by acetic acid without butylamine treatment, the framework nanostructure cannot be obtained (Fig. S8). The XRD pattern of the sample at this stage exhibits a dominated *fcc*-Pt phase (Fig. 1f). The Bi 4*f* peaks (Fig. 1g) shift to lower binding energy compared with those of butylamine-treated M/PtBi nanoplates due to the removal of Bi species [48, 49]. Specially, the remaining *hcp* PtBi intermetallic phase is observed, which is crucial to the subsequent formation of a suitable amount Bi adatoms on the surface. Due to the segregation of readily oxidized Bi atoms, the complete transformation of inner PtBi intermetallic is achieved by annealing at 200 °C in the air for 1 h [38], as indicated by the vanished *hcp* PtBi diffraction peaks. After modification, the diameter of the constructed M/Pt-BiO_x_ nanostructure decreased to ~ 13.9 nm (Fig. S9a), which is conducive to exposing more active sites. There are no corresponding diffraction patterns of Bi-related species, and the oxidized Bi (Bi^3+^: 96.0%) is dominant as revealed by the X-ray photoelectron spectrum (XPS) of Bi 4*f* (Fig. 1g), indicating the formation of amorphous Bi adatoms. The presence of metallic Bi (Bi^0^: 4.0%) could be ascribed to the residual Bi atoms buried in the Pt edges. These results could be further verified by the retained trace amount of Bi (6.7%) in the M/Pt-BiO_x_ nanoframes after cyclic voltammetry (CV) sweeping in 0.5 M H_2_SO_4_ electrolyte. According to the XPS in-depth analyses of the as-obtained M/Pt-BiO_x_ nanoframes (Fig. 1h), the Bi 4*f*_7/2_ peak appears quickly and then its intensity decreases with the increase of sputtering time, while the intensities of Pt 4*f*_7/2_ peaks increase gradually. This result further indicates the presence of Bi adatoms on Pt edges throughout the whole M/Pt-BiO_x_ nanoframes. As shown in Figs. S5d and S10, the XPS fitting results reveal the dominant presence of metallic Pt and M in M/Pt-BiO_x_.

Thus, the M/Pt-BiO_x_ nanoframes composed of dilute M/Pt ultrathin edges and surrounding BiO_x_ adatoms (IV, Fig. 1e) were successfully constructed by taking advantage of the intrinsically isolated atoms on the PtBi intermetallic along with the adjustable Bi atoms under alkaline, acidic, and oxidizing conditions sequentially. The average diameter of M/Pt-BiO_x_ edge is ~ 1.5 nm (Fig. S9b). More generally, this method could be applicable as a universal approach to fabricating well-defined multimetallic electrocatalysts with sophisticated nanostructures at the atomic-level.

**Fig. 2:**
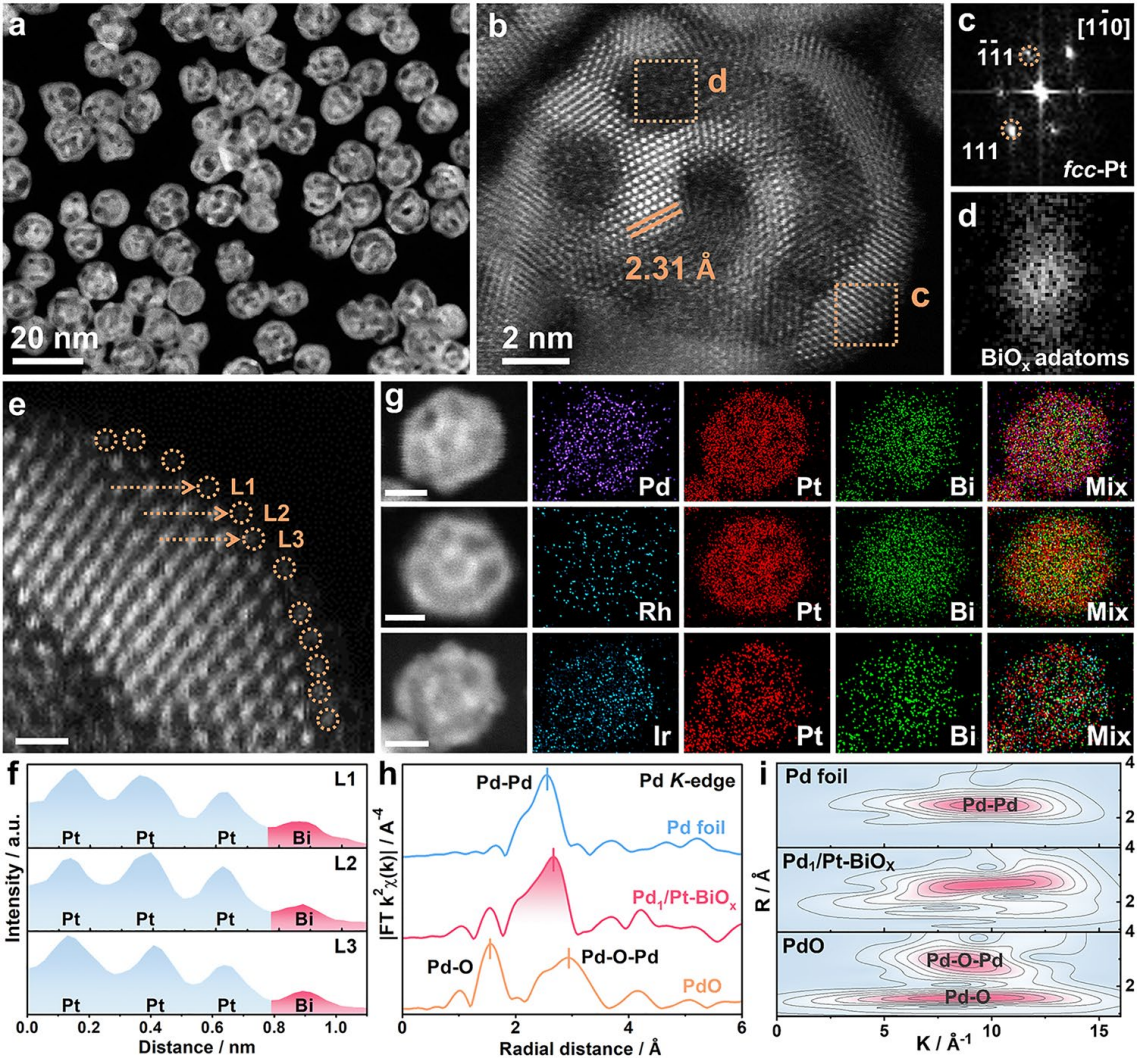
**a–g** Typical HAADF-STEM image of M/Pt-BiO_x_. **c, d** The corresponding FFT patterns of the selected regions marked in the aberration-corrected HAADF-STEM image shown in **b**. **e** A typical HAADF-STEM image showing the Pt-BiO_x_ adatoms surface. Scale bar: 0.5 nm. **f** The intensity profiles taken along the L1, L2, and L3 lines shown in **e**. **g** EDX mapping images of Pd_1_/Pt-BiO_x_, Rh/Pt-BiO_x_, and Ir/Pt-BiO_x_. Scale bars: 5 nm. **h** EXAFS spectra and **i** WT for the Pd *K*-edge EXAFS signals of Pd_1_/Pt-BiO_x_, Pd foil, and PdO powders

The HAADF-STEM images in Fig. 2a–g reveal the uniformly dispersed M/Pt-BiO_x_ heterostructures with different Z-contrasts. As shown by the aberration-corrected HAADF-STEM image in Fig. 2b, the unique heterostructures are composed of ultrathin crystalline edges and amorphous domains. The lattice spacings in the representative region with high-crystalline (as marked by c) were measured to be 2.31 Å, in consistency with that of *fcc*-Pt (111). However, in a representative region with low Z-contrast (as marked by d), atoms are arranged without long-range order. In addition, the bright spots (Fig. 2c) and diffusion ring (Fig. 2d) in the corresponding selected-area fast Fourier transform (FFT) patterns confirm the crystalline and amorphous phases, respectively [48]. In the enlarged HAADF-STEM image of a typical edge in Fig. 2e, some lower Z-contrast dots are resolved on both the outermost surface and inner surface of *fcc*-Pt ultrathin edges. Figure 2f displays the corresponding atomic arrangement of Pt/Bi atoms in this HAADF-STEM image, based on the detailed analyses of the intensity profiles of these different sites on the surface. For example, the intensity profiles of L1, L2, and L3 illustrate the typical arrangement of Pt–Pt-Bi atoms, which indicates the presence of Bi adatoms on Pt surface. The EDX mapping images in Fig. 2g indicate the framework-like distribution of Pt atoms, the atomic distribution of Pd/Ir/Rh atoms along with Pt signals, and the filling of Bi atoms in these M/Pt-BiO_x_ heterostructures. The element ratios are Pt/Bi/Pd = 70.6/25.4/4.0; Pt/Bi/Rh = 77.9/20.2/1.9; and Pt/Bi/Ir = 72.3/25.2/2.5 according to ICP-MS, similar to that of EDX results (Fig. S11). Thus, the amorphous domains could be ascribed to the three-dimensional overlapped Bi adatoms under the HAADF-STEM projection. These results indicate that the as-developed method could serve as a general strategy for the fabrication of dilute M/Pt alloy-BiO_x_ adatoms surface (Fig. S12).

Considering that Pt and Pd are the most active monometallic electrocatalysts toward EOR, the X-ray absorption near-edge structure (XANES) and extended X-ray absorption fine structure (EXAFS) analyses were performed on the Pd_1_/Pt-BiO_x_ nanoframes as a representative. Figure S13a shows the Pd *K*-edge of Pd_1_/Pt-BiO_x_ catalyst located at the range of absorption energy between Pd foil and PdO, indicating that the valence state of Pd was between 0 and + 2. Then, the average oxidation state of Pd was calculated as approximately + 1.46 based on the derivative of the XANES spectra (Fig. S13b), implying the existence of multiple coordination environments of Pd. The Fourier transform EXAFS (FT-EXAFS) spectrum in Fig. 2h reveals that the positions of the peaks in Pd_1_/Pt-BiO_x_ at around 1.6 and 2.6 Å correspond to Pd–O bond and Pd–Pt bond, respectively. In addition, there is no obvious peak observed at the position of ≈2.5 Å (for Pd–Pd coordination in metallic Pd) or ≈3.0 Å (for Pd–O-Pd coordination in PdO) in Pd_1_/Pt-BiO_x_. Furthermore, wavelet transform EXAFS (WT-EXAFS) analyses were conducted to provide accurate resolution in *k* and *R* space. The WT counter plots of Pd_1_/Pt-BiO_x_ present a maximum signal at ≈10.0 Å^−1^ probably attributed to the Pd–Pt bonds and a different signal distribution than those of metallic Pd and PdO (Fig. 2i), and their maximum signals are located at around 9.6 Å^−1^ and around 8.7 Å^−1^, respectively, being consistent with the Pd *K*-edge FT-EXAFS spectrum analyses. These results confirm that Pd atoms in Pd_1_/Pt-BiO_x_ are uniformly distributed in the form of single-atom species, and stabilized in the lattice of Pt [50, 51].

**Fig. 3:**
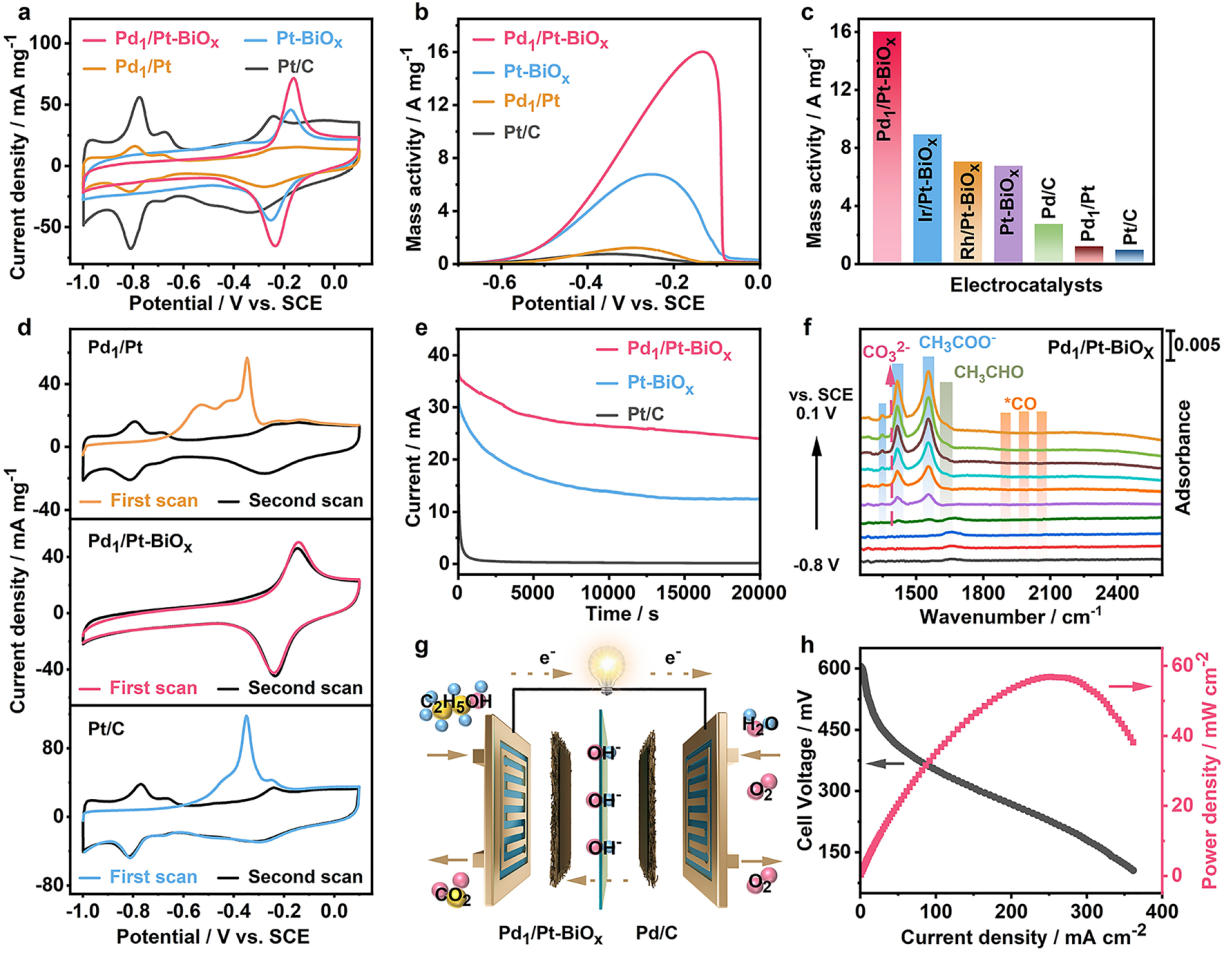
**a** CV curves of different electrocatalysts recorded in Ar-saturated 1.0 M KOH. **b** Positive-going CVs of different electrocatalysts recorded at a scan rate of 50 mV s^−1^ in Ar-saturated 1.0 M KOH + 1.0 M C_2_H_5_OH. **c** Comparisons on the mass activities between different electrocatalysts in Ar-saturated 1.0 M KOH + 1.0 M C_2_H_5_OH. **d** CO-stripping curves of Pd_1_/Pt-BiO_x_, Pd_1_/Pt, and Pt/C. **e** Chronoamperometry curves of different electrocatalysts recorded at the potential of -0.4 V vs. SCE. **f** In situ FTIR spectra of Pd_1_/Pt-BiO_x_. **g, h** Schematic illustration and power density curve of a DEFC assembled by using Pd_1_/Pt-BiO_x_ as anodic electrocatalyst and commercial Pd/C as cathodic electrocatalyst

The electrocatalytic properties of M/Pt-BiO_x_ were systemically evaluated toward EOR and DAFCs. As shown in Figs. 3a and S14a, compared with Pt/C, the hydrogen adsorption/desorption (− 1.0 to − 0.6 V vs. SCE) characteristics are negligible in the CV curves of M/Pt-BiO_x_ and Pt-BiO_x_, due to the presence of BiO_x_ adatoms on their Pt surfaces [30]. As shown by the EOR polarization curves recorded in Ar-saturated 1.0 M KOH + 1.0 M C_2_H_5_OH (Figs. 3b and S14b), Pt-BiO_x_ shows a high mass activity of 6.77 A mg^−1^_Pt_ at the peak potential, which is 6.9 times higher than that of Pt/C electrocatalyst (0.98 A mg^−1^_Pt_) [46]. By introducing a trace amount of Pd, the EOR activity is significantly enhanced than that of Pt-BiO_x_. However, after tuning the proportion of Pd from 3.3% to 9.2%, no significant change on EOR performance is observed compared with Pd_1_/Pt-BiO_x_ (Fig. S15). As shown in Fig. 3c and Table S2, Pd_1_/Pt-BiO_x_ exhibits the highest peak mass activity of 16.01 A mg^−1^_Pt+Pd_, which is 2.4, 5.8, and 16.4 times higher than those of Pt-BiO_x_, Pd/C, and Pt/C electrocatalysts, respectively [52]. This result indicates the synergetic effect of Pd_1_ and Pt-BiO_x_ heterointerfaces in boosting electrocatalytic oxidation of ethanol on Pt [53]. On the other hand, Ir/Pt-BiO_x_ (8.92 A mg^−1^_Pt+Ir_), Rh/Pt-BiO_x_ (7.05 A mg^−1^_Pt+Rh_), and Ru/Pt-BiO_x_ (7.59 A mg^−1^_Pt+Ru_) show no evident increase in their mass activities compared with that of Pt-BiO_x_. These results illustrate the importance of engineering unique active ensembles toward specific applications [54, 55]. The etching of BiO_x_ could be achieved by CV sweeping Pd_1_/Pt-BiO_x_ in Ar-saturated 0.5 M H_2_SO_4_ electrolyte for 400 cycles at the potential ranging from − 0.25 to 0.8 V vs. SCE, forming Pd_1_/Pt. After this treatment, the Bi content decreased sharply from the initial value of 25.4% to 6.7% (Fig. S16), and the typical peaks in the CV curve of Pd_1_/Pt are similar to those of Pt/C (Fig. 3a). In addition, after removing BiO_x_ by soaking Pd_1_/Pt-BiO_x_ in 0.5 M H_2_SO_4_ at room temperature for 30 min (to avoid loss of the active site of Pt), the electrochemical test result is similar, showing a sharp decline in mass activity of Pd_1_/Pt-BiO_x_ toward EOR (Fig. S17). By comparing Figs. 2a and S18, BiO_x_ adatoms with low Z-contrast disappear, and the presence of Pt nanoframes in the initial Pd_1_/Pt-BiO_x_ nanostructure is observed. These results demonstrate that the beneficial BiO_x_ adatoms that are removed. As an oxyphilic metal, Bi species enables the formation of adsorbed hydroxyl species (-OH_ad_) at lower potentials, which can mitigate the poisoning of CO_ads_ species on Pt surface [56]. As a result, the peak mass activity of Pd_1_/Pt-BiO_x_ catalyst toward EOR decreased dramatically to 1.21 A mg^−1^_Pt+Pd_, revealing the importance of surrounding BiO_x_ adatoms on the *fcc*-Pt edges in boosting EOR electrocatalysis [30].

Figure 3d shows the CO-stripping curves of Pt/C, Pd_1_/Pt-BiO_x_, and Pd_1_/Pt electrocatalysts. The noticeable CO oxidation features are observed in the Pd_1_/Pt and Pt/C electrocatalysts, but are absent in Pd_1_/Pt-BiO_x_ [57, 58]. The result demonstrates that the BiO_x_ adatoms are crucial for resolving the typical CO-poisoning issues on Pt [59, 60]. It is worth noting that Pd_1_/Pt and Pt/C electrocatalysts exhibit comparable mass activities and CO oxidation features, illustrating the negligible effect of residual Bi atoms buried in Pt edges and the limited promotion of Pd atoms without mitigating the CO-poisoning issues by BiO_x_ adatoms. On the other hand, the durability could be greatly affected by the anti-poisoning ability [28]. As a result, Pt/C catalyst suffers from a rapid drop in current density to nearly 0 mA cm^−2^, as shown by the chronoamperometry (CA) measurements at − 0.4 V vs. SCE (Fig. 3e). In contrast, the highly active Pd_1_/Pt-BiO_x_ and Pt-BiO_x_ electrocatalysts retain 58.2% and 37.6% of their initial values after CA measurements at − 0.4 V vs. SCE for 20,000 s, respectively. In addition, the Pd_1_/Pt-BiO_x_ exhibits an excellent structural stability in alkaline electrolyte, as demonstrated by the well-maintained composition (Pt/Bi/Pd = 77.0/19.9/3.1) after running CA for 20,000 s (Figs. S19 and S20).

The in situ Fourier transform infrared spectroscopy (FTIR) spectra in Fig. 3f show the behavior of absorbance on Pd_1_/Pt-BiO_x_ electrocatalyst in 1.0 M KOH + 1.0 M CH_3_CH_2_OH. The positive- and negative-going peaks represent the gain and loss of species at the sample potential, respectively. For Pd_1_/Pt-BiO_x_, the ethanol oxidation begins from the potential of ~ − 0.8 V vs. SCE, as evidenced by the positive-going peaks representing the gain of acetaldehyde (~ 1631 cm^−1^) and acetate (1347, 1417, and 1554 cm^−1^). However, CO_3_^2−^ with the IR band at 1390 cm^−1^ overlaps with CH_3_COO^−^ at ~ 1417 cm^−1^, leading to the difficulty in determining the production of CO_2_. The Ba^2+^ will react with CO_3_^2−^ to generate precipitation of BaCO_3_, thus the amount of CO_3_^2−^ generated can be measured by weighing the precipitate [61]. As a result, the C1 pathway (CO_2_/CO_3_^2−^) FE of Pd_1_/Pt-BiO_x_ toward EOR in alkaline electrolyte can be obtained (~ 5.3%) according to the mass of BaCO_3_ precipitation. It is worth noting that the CO signals are observed on the active sites of Pt/C at considerably low potentials (Fig. S21) [62].

As for the Pd_1_/Pt-BiO_x_, the CO signals are extremely weak with increased potentials, indicating the absence of CO poisoning. In addition, a home-made DEFC is assembled by using Pd_1_/Pt-BiO_x_ (anodic electrocatalyst), Pd/C (cathodic electrocatalyst), and KOH-doped polybenzimidazole membrane (Fig. 3g). Figure 3h shows the polarization and power density curve of the as-obtained DEFC, recorded by using O_2_ as cathodic gas feed, 4.0 M CH_3_CH_2_OH + 6.0 M KOH electrolyte as anodic fuel feed, and the cell temperature of 60 °C. As a result, the peak power density on Pd_1_/Pt-BiO_x_ reaches 56.7 mW cm^2^, higher than that of a DEFC (28.9 mW cm^2^) assembled by commercial Pt/C and Pd/C catalysts (Fig. S22) and the as-reported values achieved by developing EOR electrocatalysts [63–66].

Besides, Pt-BiO_x_ could also enhance the methanol oxidation reaction (MOR) electrocatalysis in alkaline electrolyte. As shown in Figs. S23 and S24, Pt-BiO_x_ exhibits a high mass activity of 12.07 A mg^−1^_Pt_ toward MOR, which is 6.5 times higher than that of Pt/C (1.85 A mg^−1^_Pt_) [27]. Specially, Rh/Pt-BiO_x_ shows the highest mass activity of 15.31 A mg^−1^_Pt+Rh_, 8.3 times higher than that of Pt/C. The negative shift of methanol oxidation potential on Rh/Pt-BiO_x_ further indicates more rapid MOR kinetics than on other electrocatalysts (Fig. S23a), indicating the synergetic effect of Rh/Pt dilute alloy and BiO_x_ adatoms in boosting MOR. It is also observed that unlike Pt-BiO_x_, Ir/Pt-BiO_x_ (12.08 A mg^−1^_Pt+Ir_) and Pd_1_/Pt-BiO_x_ (10.62 A mg^−1^_Pt+Pd_) show no increase in their mass activities (Fig. S24). These results illustrate the importance of engineering unique catalyst surface toward specific applications [54, 55]. Similarly, by sweeping from − 0.25 to 0.8 V vs. SCE in Ar-saturated 0.5 M H_2_SO_4_ electrolyte for 400 cycles at a scan rate of 100 mV s^−1^, the mass activity of Rh/Pt-BiO_x_ toward MOR dropped dramatically to 1.49 A mg^−1^_Pt+Rh_, further demonstrating the critical role of surrounding BiO_x_ in enhancing MOR. As shown in Fig. S23b, c, Rh/Pt-BiO_x_ exhibits superior anti-poisoning ability and durability compared with Pt/C, as indicated by the eliminated features of electrochemical CO oxidation and the highly retained current density (62.0%) after long-term CA measurements at − 0.4 V vs. SCE.

**Fig. 4:**
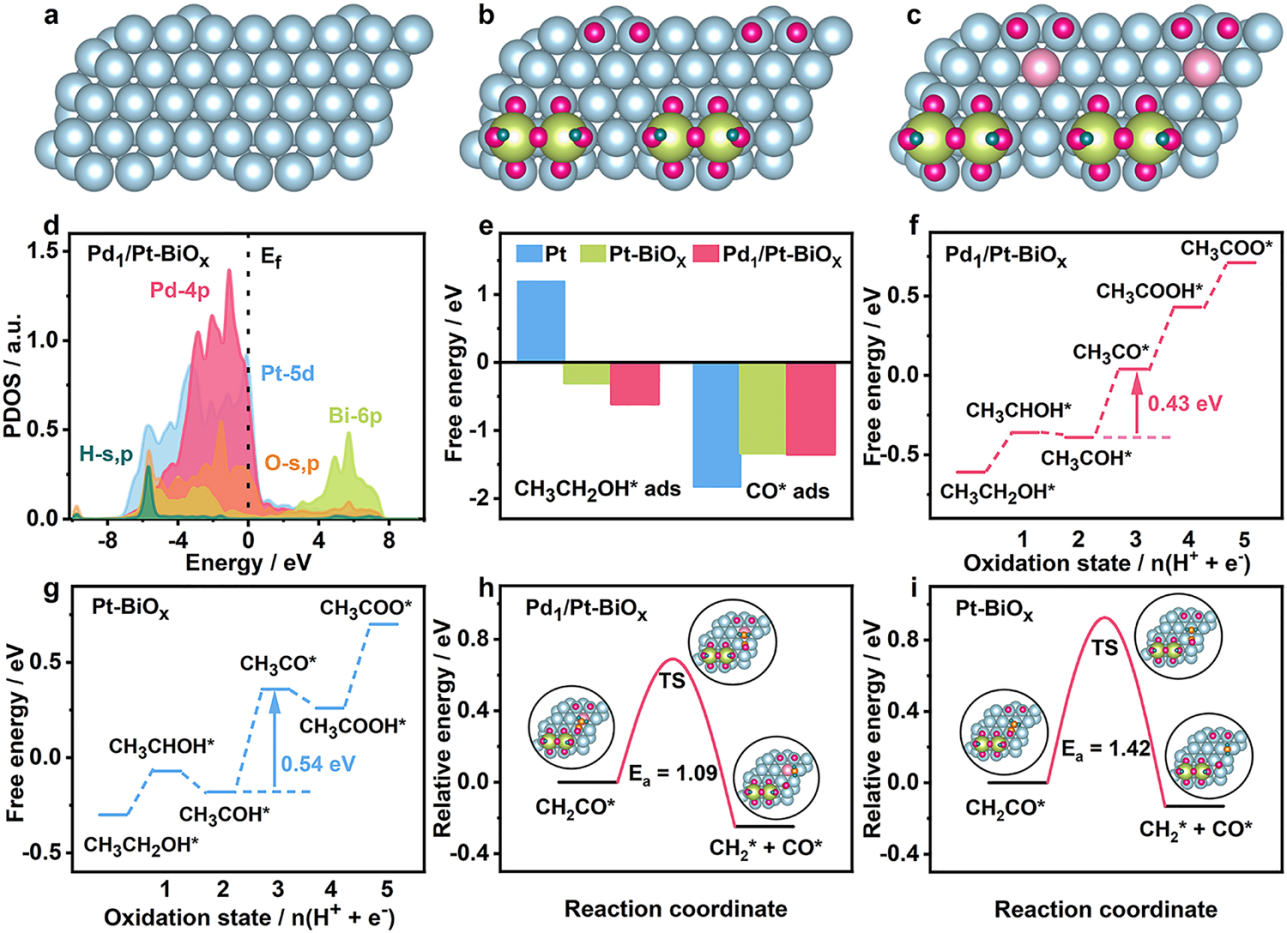
**a–c** Structural models of Pt, Pt-BiO_x_, and Pd_1_/Pt-BiO_x_ from the top view. **d** PDOS of Pd_1_/Pt-BiO_x_. **e** Adsorption comparison of CH_3_CH_2_OH and CO on Pt, Pt-BiO_x_, and Pd_1_/Pt-BiO_x_. **f, g** Potential energy diagram for EOR via the C2 pathway on Pd_1_/Pt-BiO_x_ and Pt-BiO_x_. **h, i** Energy profiles for breaking the C–C bond of CH_2_CO intermediate on Pd_1_/Pt-BiO_x_ and Pt-BiO_x_. Blue, herb green, dark green, purple, and pink spheres represent Pt, Bi, H, O, and Pd atoms, respectively

DFT calculations are further introduced to explore the mechanism of the improved EOR performance on Pd_1_/Pt-BiO_x_. Figure 4a–c shows the surface structural models for Pt, Pt-BiO_x_, and Pd_1_/Pt-BiO_x_, respectively. For the relative position of Pd_1_ in Pt-BiO_x_, we established three adsorption models, and the corresponding structures are shown in Fig. S25. Among them, when Pd_1_ and BiO_x_ are not adjacent (Fig. S25a), the corresponding structure of ethanol adsorption energy is the lowest (ΔG_ads_ = − 0.61 eV). Therefore, we finally chose this structural model for DFT calculations. In addition, during the EOR process in alkaline electrolyte, Bi adatoms can be oxidized into bismuth oxyhydroxide, resulting in the formation of the BiO_x_(OH)_y_-Pt inverse interfaces. The detailed electronic structures of Pd_1_/Pt-BiO_x_ are demonstrated through the projected partial density of states (PDOS) (Figs. 4e and S26). Notably, the Pt-5*d* orbitals display an evident peak at − 2.68 eV and the high d-band center at − 2.49 eV demonstrates the high electroactivity for the EOR process. It is noted that O-*s*, *p* orbitals show a good overlapping with Bi orbitals, supporting the strong binding and efficient site-to-site electron transfer of the electrocatalyst. Bi-6*p* orbitals exhibit broad coverage crossing the E_f_ with the main contribution at the anti-bonding orbitals, which facilitates the binding of intermediates on the surface. Meanwhile, the high electron density of Bi-*s*, *p* orbitals near E_f_ further contributes to the electron transfer between the electrocatalyst surface and the adsorbed intermediates. Furthermore, we also investigated the EOR reaction trend from the free energetic perspective. Figures 4e and S27 show that ethanol strongly adsorbs on the surface of Pt after the introduction of BiO_x_(OH)_y_, with the Gibbs free adsorption energies (ΔG_ads_) of − 0.30 and − 0.61 eV for Pt-BiO_x_ and Pd_1_/Pt-BiO_x_, respectively. At the same time, higher adsorption energies of CO on Pt-BiO_x_ (− 1.33 eV) and Pd_1_/Pt-BiO_x_ (− 1.35 eV) than that on Pt (− 1.83 eV) reveal much weaker bonding strength between CO and Pt atoms, indicating that BiO_x_(OH)_y_ on Pt surface has the effect of suppressing CO-poisoning during EOR. These results demonstrate that the introduction of BiO_x_(OH)_y_ could enhance the adsorption of reactant and reduce the CO toxicity, thus improving the EOR performance of Pd_1_/Pt-BiO_x_ and Pt-BiO_x_ electrocatalysts.

We studied the role of Pd_1_: In the presence of Pd_1_, the oxidation of the determining step of EOR requires (CH_3_CHOH* to CH_3_COH*) 0.43 eV on Pd_1_/Pt-BiO_x_ (Figs. 4f and S28), while 0.54 eV is required on Pt-BiO_x_ (Figs. 4g and S29). For C1 pathway, the energy barrier for breaking CH_2_CO* to CH_2_* and CO* is 1.09 eV on Pd_1_/Pt-BiO_x_, while 1.42 eV is required on Pt-BiO_x_. These research outputs collectively confirm that introducing BiO_x_(OH)_y_ and doping Pt surface with Pd_1_ synergistically contribute to enhancing EOR performance via improving the adsorption of reactants, enhancing the anti-toxicity ability, promoting the oxidation of key reaction intermediates, and facilitating the C–C fracture.

## Conclusion

We successfully constructed a novel M/Pt dilute alloy-BiO_x_ adatoms surface via rational design and multifactorial engineering of the fully ordered *hcp* PtBi nanoplates with intrinsically isolated Bi atoms. These unique M/Pt-BiO_x_ electrocatalysts are composed of Pt nanoframes with their ultrathin *fcc*-Pt edges being surface-modified by atomically dispersed M (*M* = Pd, Rh, or Ir) and surrounding BiO_x_ adatoms. Benefiting from the eliminated CO-poisoning on Pt-BiO_x_ surface and the optimized electrooxidation activity on dilute alloy, both Pd_1_/Pt-BiO_x_ and Rh/Pt-BiO_x_ achieve the best-performing EOR and MOR electrocatalysis, respectively, outperforming most of the as-developed Pt-based electrocatalysts. A practical DEFC with high power density is realized by deploying Pd_1_/Pt-BiO_x_ as anodic electrocatalyst. This work highlights the importance of multifactorial engineering strategy in a nanostructure at atomic-level to develop advanced and sophisticated electrocatalysts for broad applications.

## Supplementary Information

Below is the link to the electronic supplementary material.

Supplementary file1 (DOCX 14451 KB)
