# Supplementary material for: Boosting Alcohol Oxidation Electrocatalysis with Multifactorial Engineered Pd1/Pt Single-Atom Alloy-BiOx Adatoms Surface
Source: Nanomicro Lett. 2025 Mar 3;17:172. doi: 10.1007/s40820-025-01678-4 (PMC11872865; doi:10.1007/s40820-025-01678-4)

Supporting Information for

**Boosting Alcohol Oxidation Electrocatalysis with Multifactorial Engineered Pd_1_/Pt Single Atom Alloy-BiO_x_ Adatoms Surface**

Yujia Liao^1,2,†^,Wen Chen^1,†^, Yutian Ding^1, †^, Lei Xie^1,4,†^, Qi Yang^2^, Qilong Wu^3^, Xianglong Liu^1^, Jinliang Zhu^4^, Renfei Feng^5^, Xian-Zhu Fu^1^, Shuiping Luo^1,3,^*, Jing-Li Luo^1^

^1^ Shenzhen Key Laboratory of Energy Electrocatalytic Materials, Guangdong Provincial Key Laboratory of New Energy Materials Service Safety, College of Materials Science and Engineering, Shenzhen University, Shenzhen, Guangdong 518055, P. R. China

^2^ Department of Chemistry, City University of Hong Kong, Kowloon, Hong Kong 999077, P. R. China

^3^ Department of Chemistry, Southern University of Science and Technology (SUSTech), Shenzhen, Guangdong 518055, P. R. China

^4^ School of Resources, Environment and Materials, MOE Key Laboratory of New Processing Technology for Nonferrous Metals and Materials, Guangxi University, Nanning, Guangxi 530004, P. R. China

^5^ Canadian Light Source Inc., 44 Innovation Blvd., Saskatoon, S7N 0×4, SK, Canada

† Yujia Liao, Wen Chen, Yutian Ding, and Lei Xie contributed equally to this work.

* Corresponding authors. E-mail: [luosp@szu.edu.cn](mailto:luosp@szu.edu.cn) (Shuiping Luo)

**Supplementary Figures and Tables**


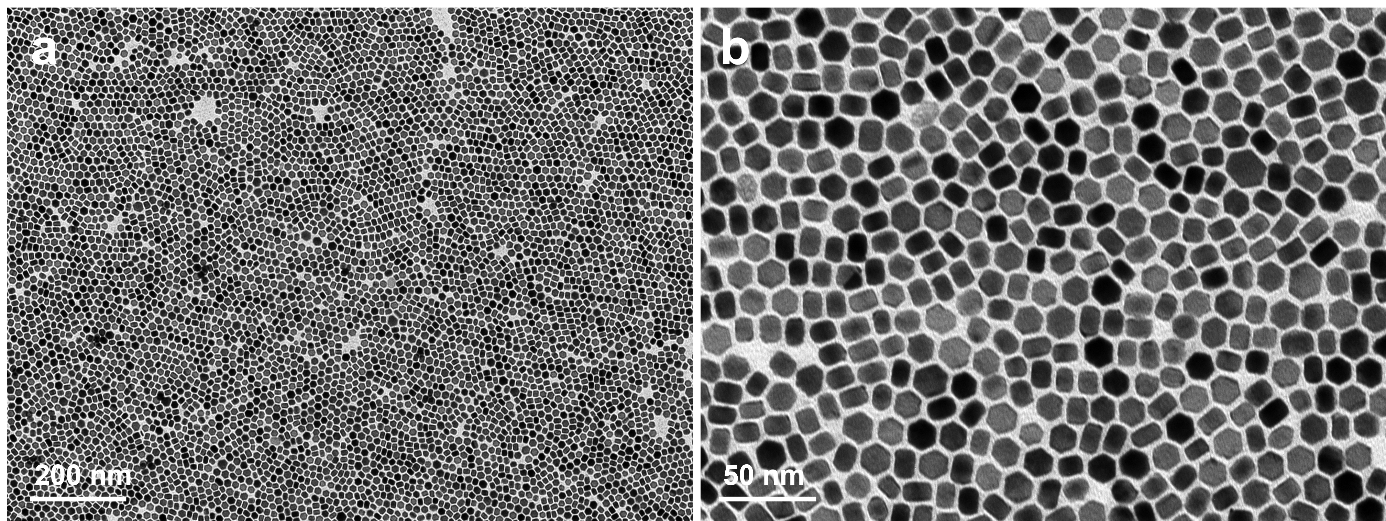


**Fig. S1** Large area TEM images of M/PtBi nanoplates

**
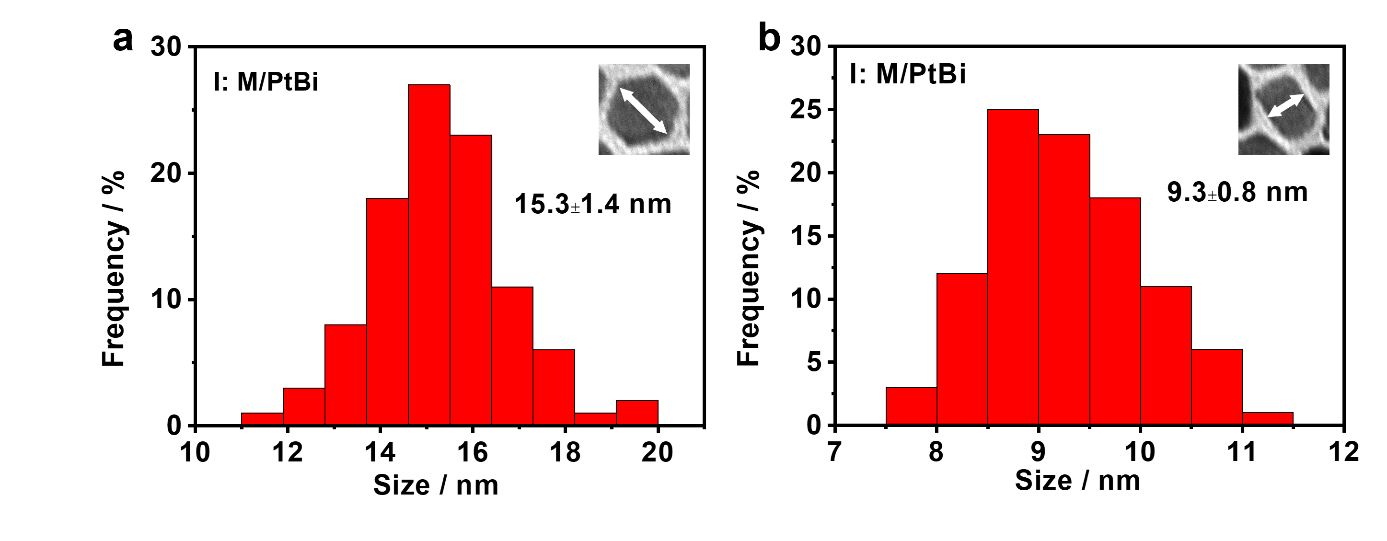
**

**Fig. S2** Diameter (**a**) and thickness (**b**) distribution of M/PtBi nanoplates


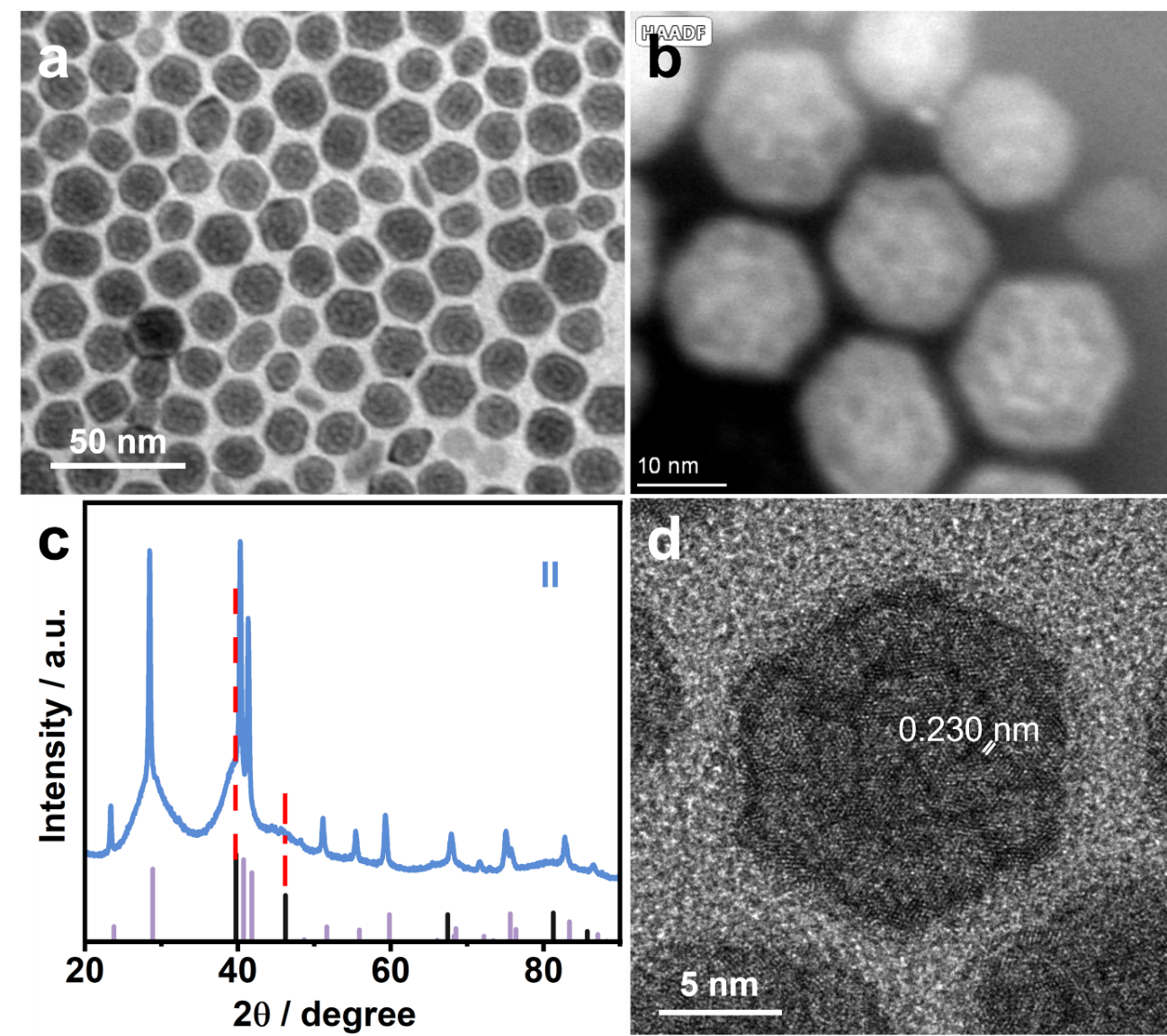


**Fig. S3** (**a**) TEM image, (**b**) HAADF-STEM image, (**c**) XRD pattern, and (**d**) high-resolution TEM image of butylamine-treated M/PtBi nanoplates (II).


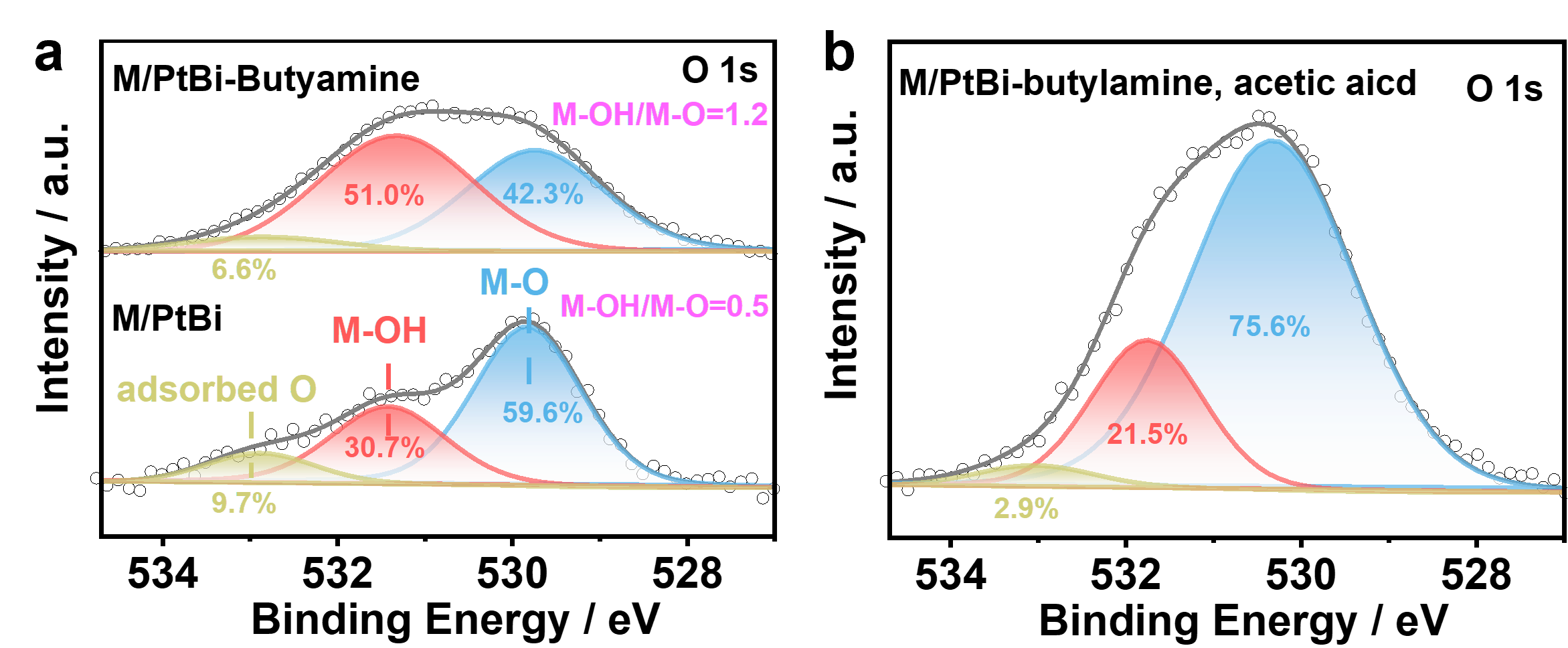


**Fig. S4** (**a**) O 1s XPS spectra of M/PtBi before (I) and after (II) treatment with butylamine. (**b**) O 1s XPS spectra of M/PtBi after treatment with butylamine and acetic acid (III). All of the spectra were calibrated by C1s peak located at 284.8 eV


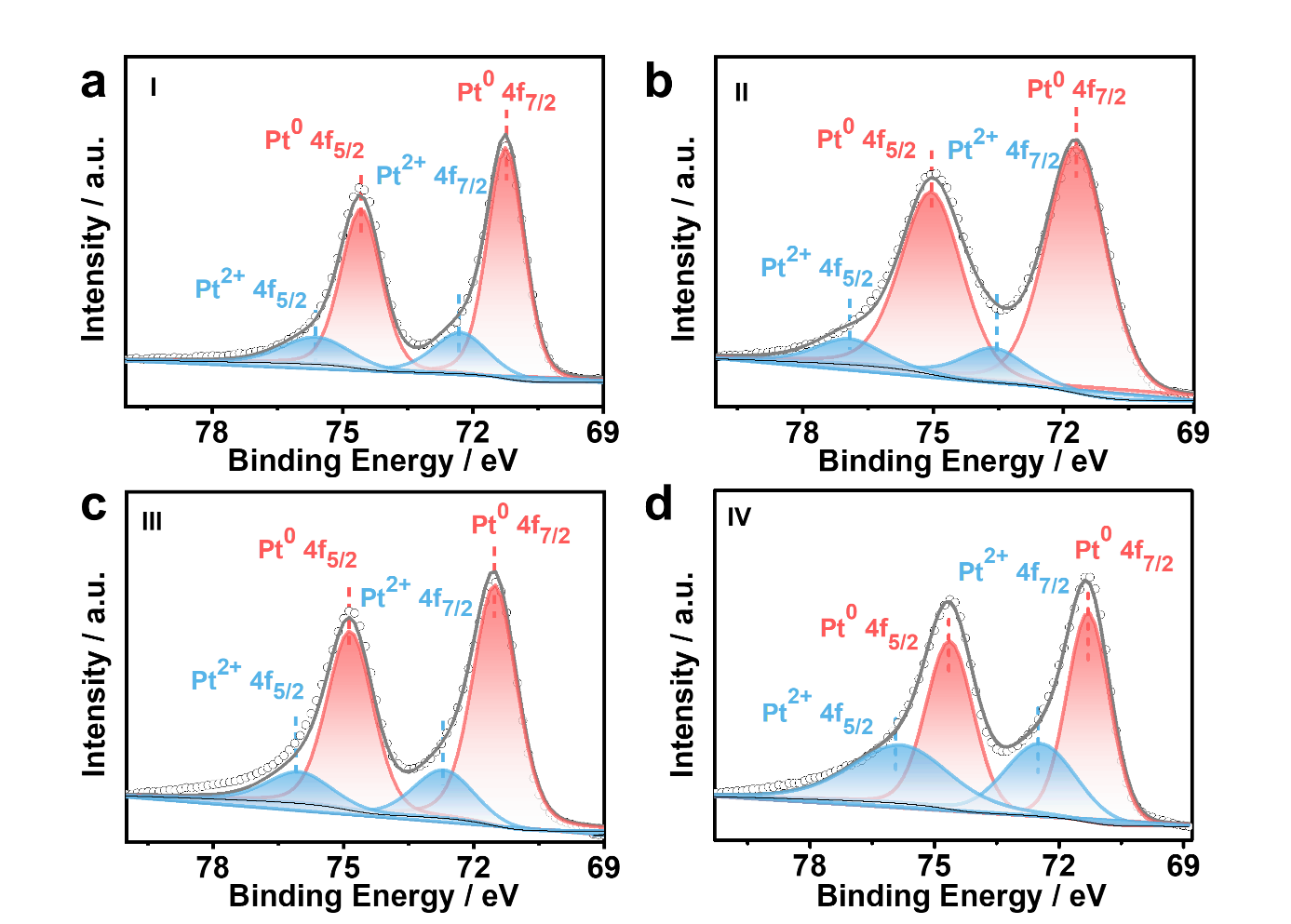


**Fig. S5** Pt 4f XPS spectra of I, II, III, and IV. All of the spectra were calibrated by C1s peak located at 284.8 eV


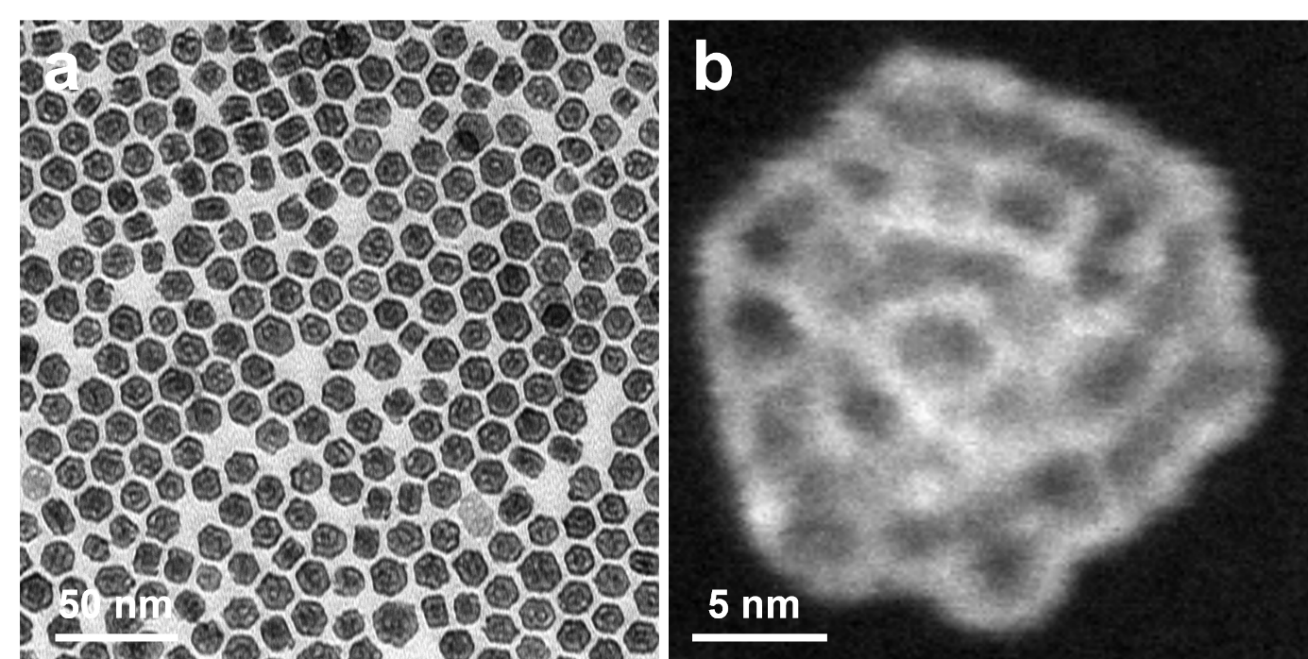


**Fig. S6** (**a**) TEM, and (**b**) HAADF-STEM images of M/PtBi after treatment with butylamine and acetic acid (III)


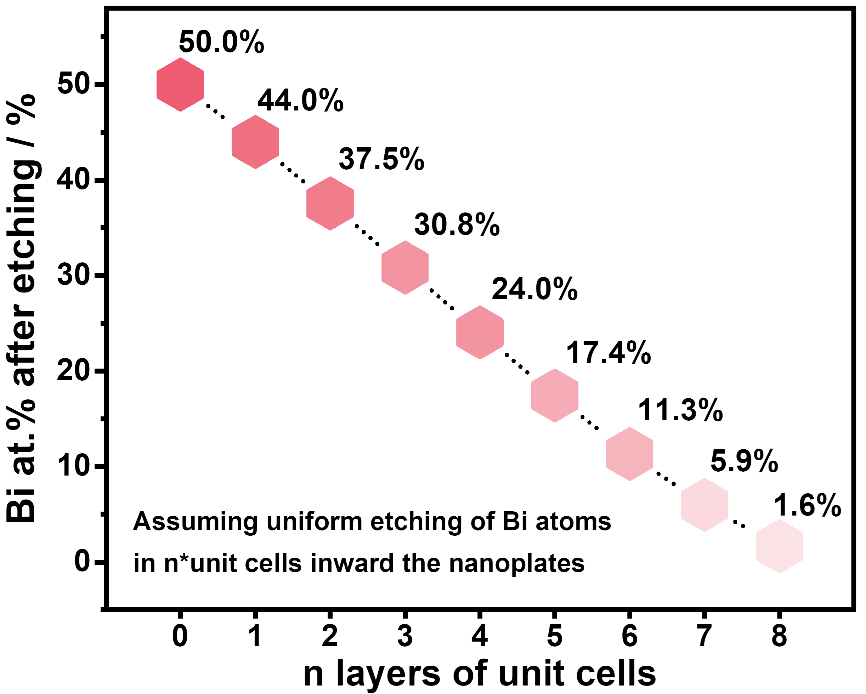


**Fig. S7** The Bi atomic percentage retained after the etching of Bi atoms in top n layers of PtBi unit cells

$$\mathbf{Bi}\boldsymbol{at. \%=}\frac{\left( \boldsymbol{15.3-0.863}\boldsymbol{n} \right)^{\boldsymbol{2}}\left( \boldsymbol{9.3-1.098}\boldsymbol{n} \right)}{\left( \boldsymbol{15.3-0.863}\boldsymbol{n} \right)^{\boldsymbol{2}}\left( \boldsymbol{9.3-1.098}\boldsymbol{n} \right)\boldsymbol{+2177.04}}$$

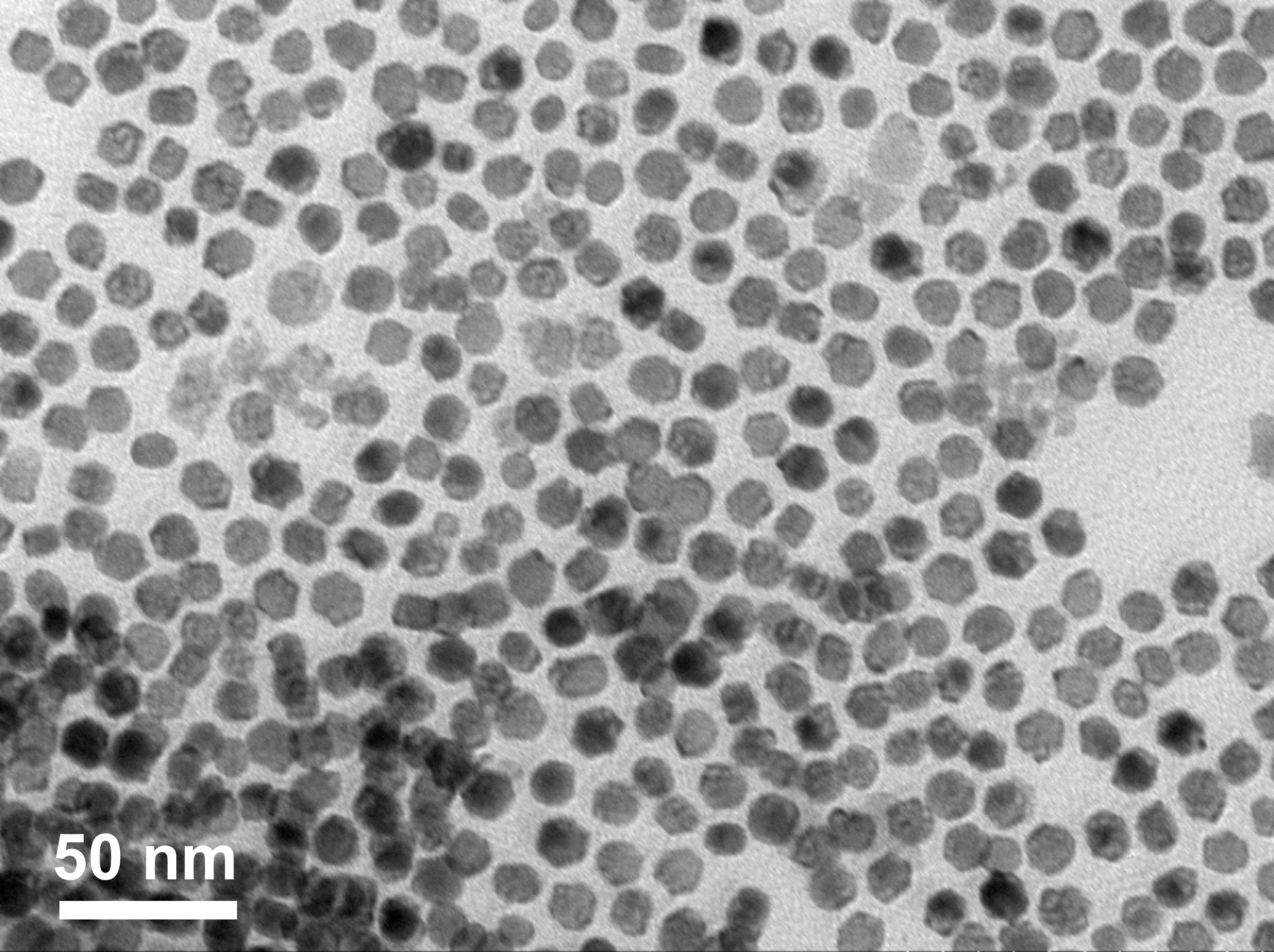


**Fig. S8** TEM image of M/PtBi nanoplates treated directly with acetic acid without butylamine

**
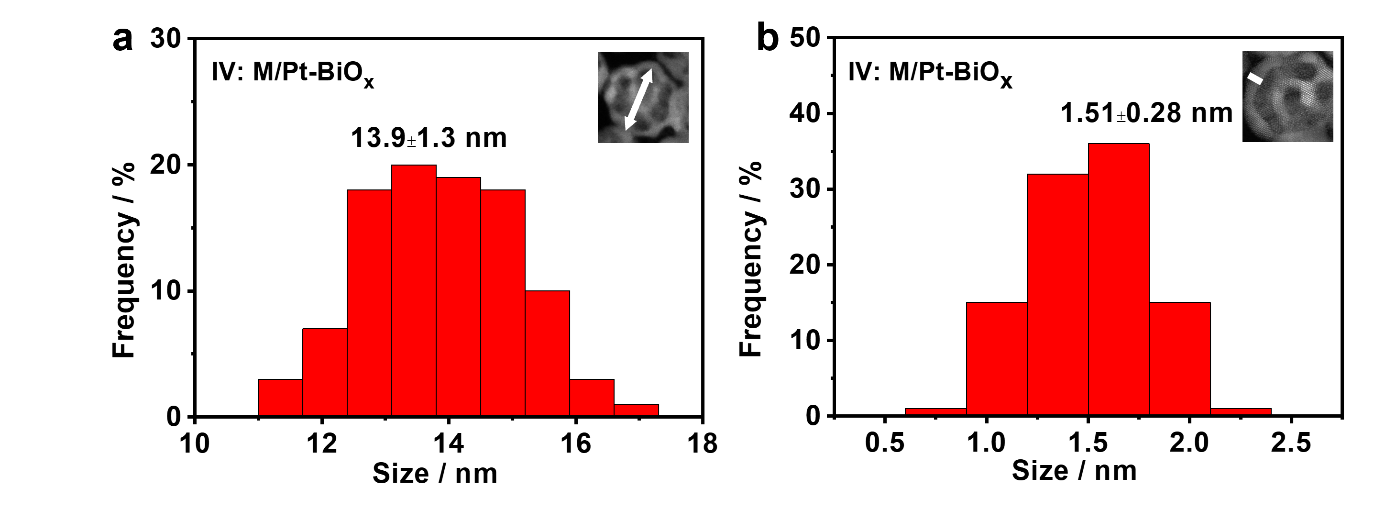
**

**Fig. S9** Diameter (**a**) and thickness (**b**) distribution of M/Pt-BiO_x_

**
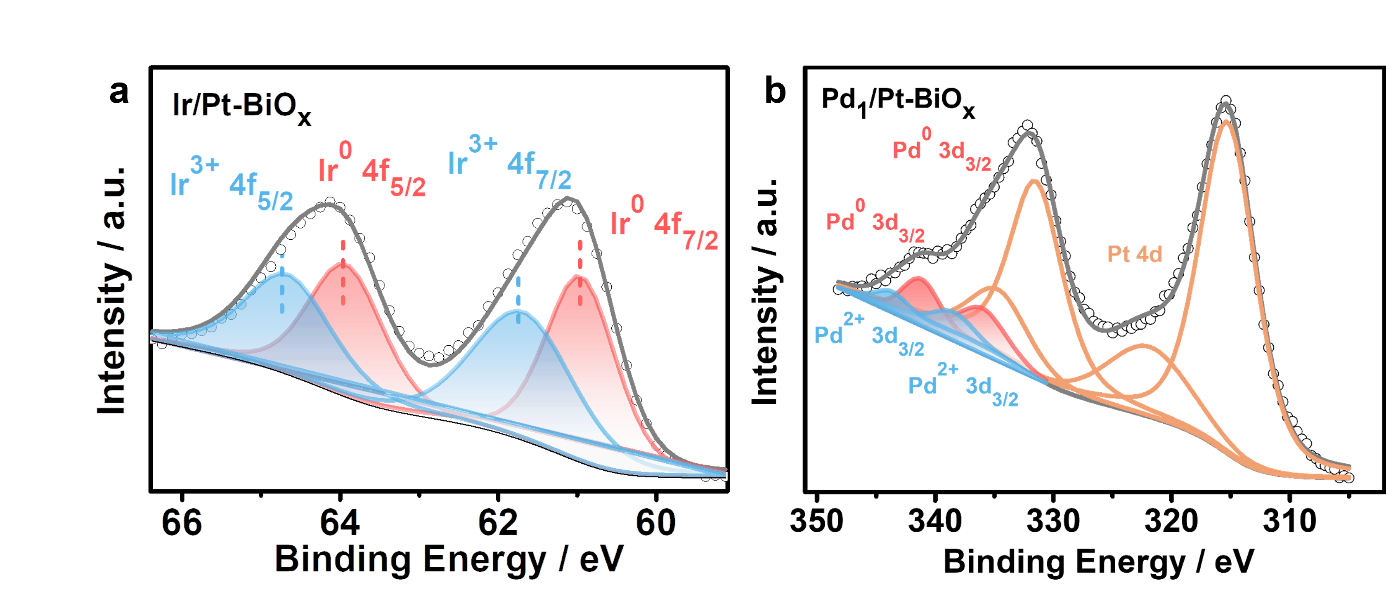
**

**Fig. S10** (**a**) Ir 4f XPS spectra of Ir/Pt-BiO_x_; (**b**) Pd 3d XPS spectrum of Pd_1_/Pt-BiO_x_. All of the spectra were calibrated by C1s peak located at 284.8 eV


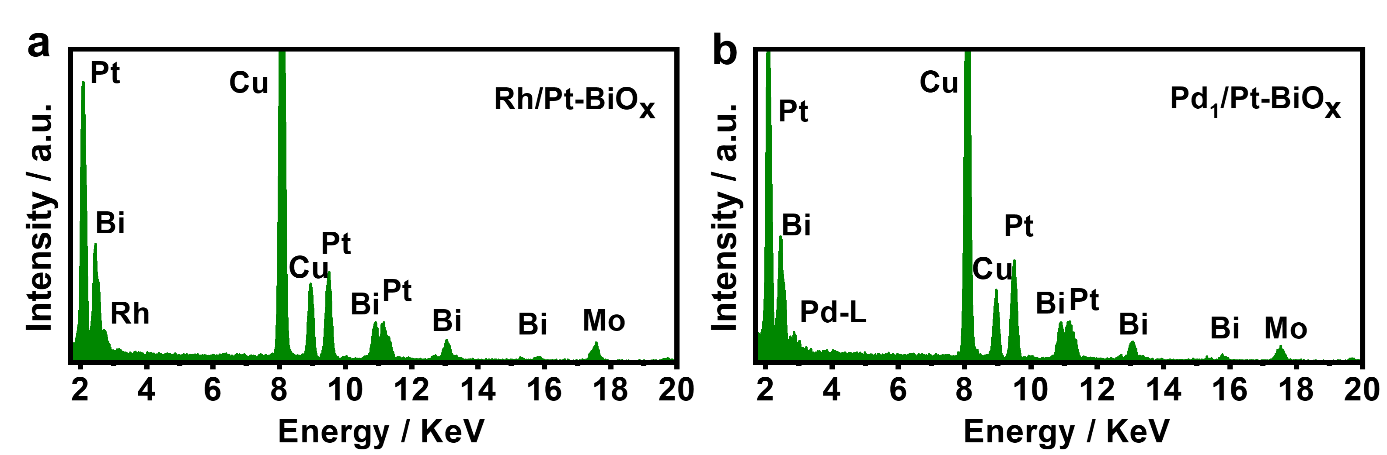


**Fig. S11** EDX spectra of (**a**) Rh/Pt-BiO_x_ and (**b**) Pd_1_/Pt-BiO_x_


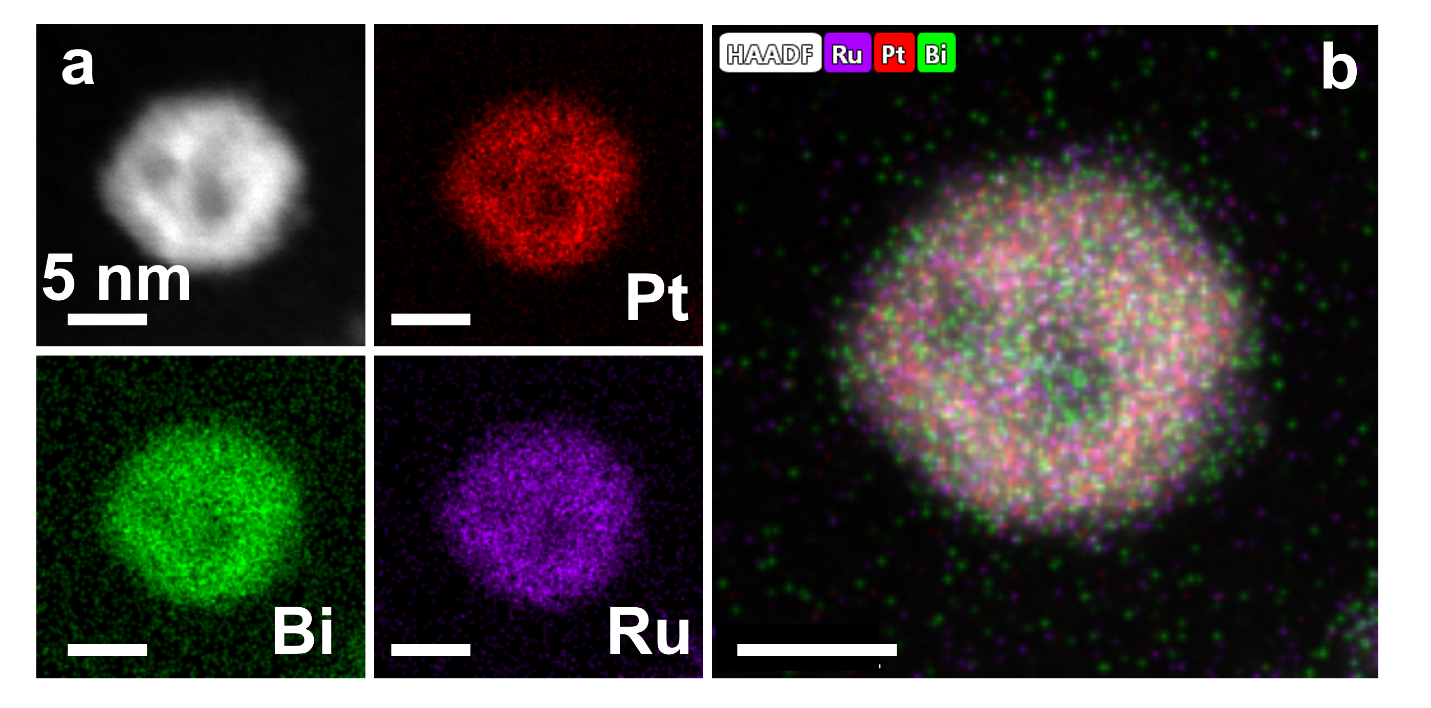


**Fig. S12** (**a**) HAADF-STEM image and corresponding EDX maps of Ru/Pt-BiO_x_. (**b**) The overlap of HAADEF-STEM image and corresponding EDX maps in (a). Scale bars: 5 nm

**
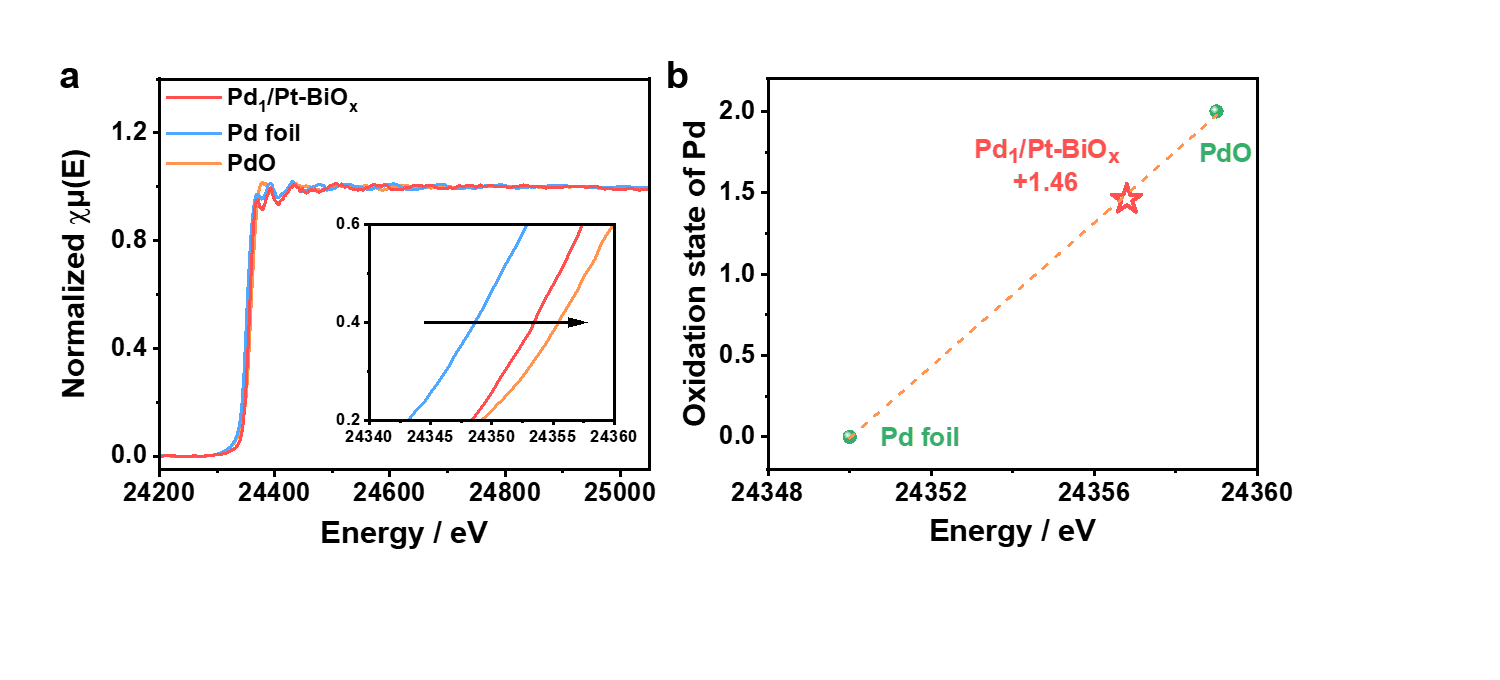
**

**Fig. S13** (**a**) Pd *K*-edge spectra of Pd_1_/Pt-BiOx, Pd foil, and PdO. (**b**) Oxidation calculations for Pd in Pd_1_/Pt-BiOx, Pd foil, and PdO

**
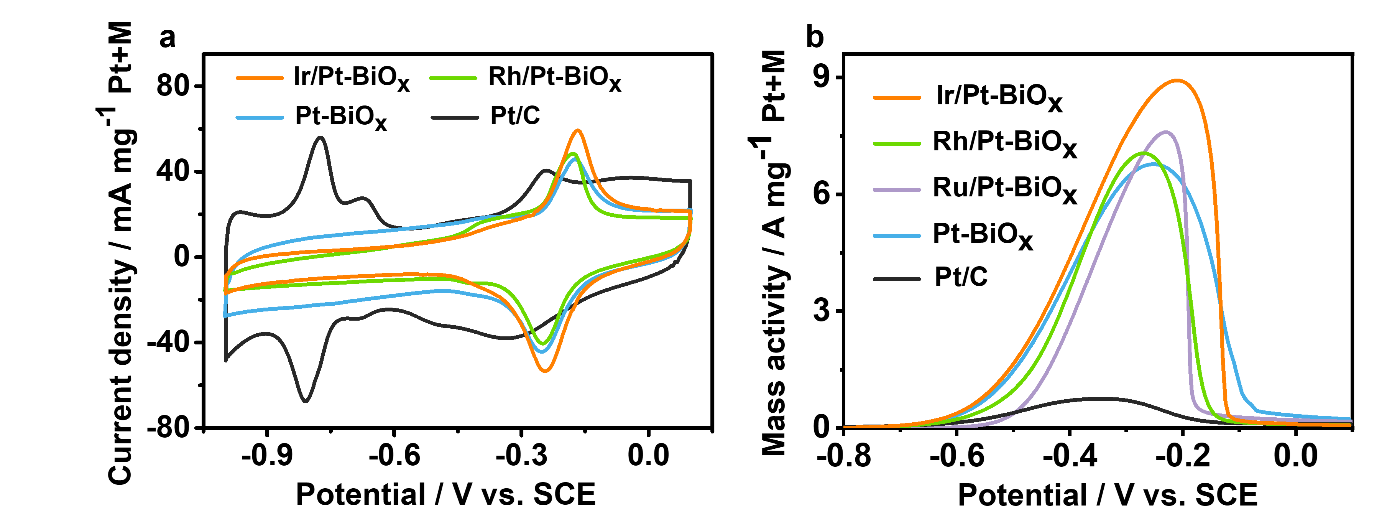
**

**Fig. S14** (**a**) CVs of Ir/Pt-BiO_x_, Rh/Pt-BiO_x_, Pt-BiO_x_, and Pt/C electrocatalysts recorded at a scan rate of 50 mV s^-1^ in Ar-saturated 1.0 M KOH electrolyte. (**b**) The positive-going EOR anodic scan polarization curves of Ir/Pt-BiO_x_, Rh/Pt-BiOx, Ru/Pt-BiOx, Pt-BiO_x_, and Pt/C electrocatalysts in Ar-saturated 1.0 M KOH + 1.0 M CH_3_CH_2_OH at a scan rate of 50 mV s^-1^


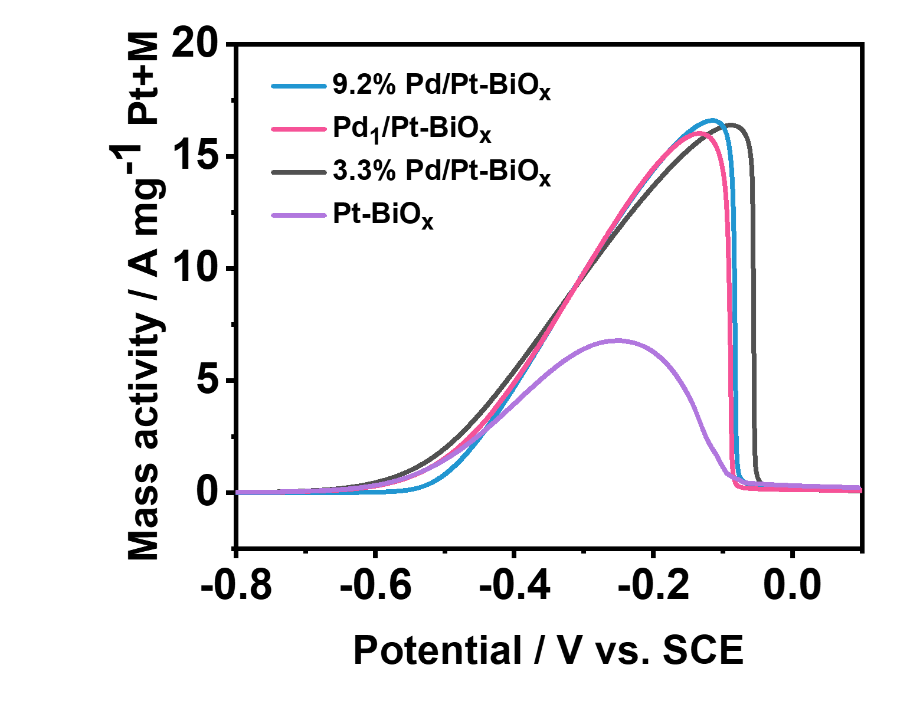


**Fig. S15** The positive-going EOR anodic scan polarization curves of Pd/Pt-BiO_x_ with different Pd contents in Ar-saturated 1.0 M KOH + 1.0 M CH_3_CH_2_OH at a scan rate of 50 mV s^-1^


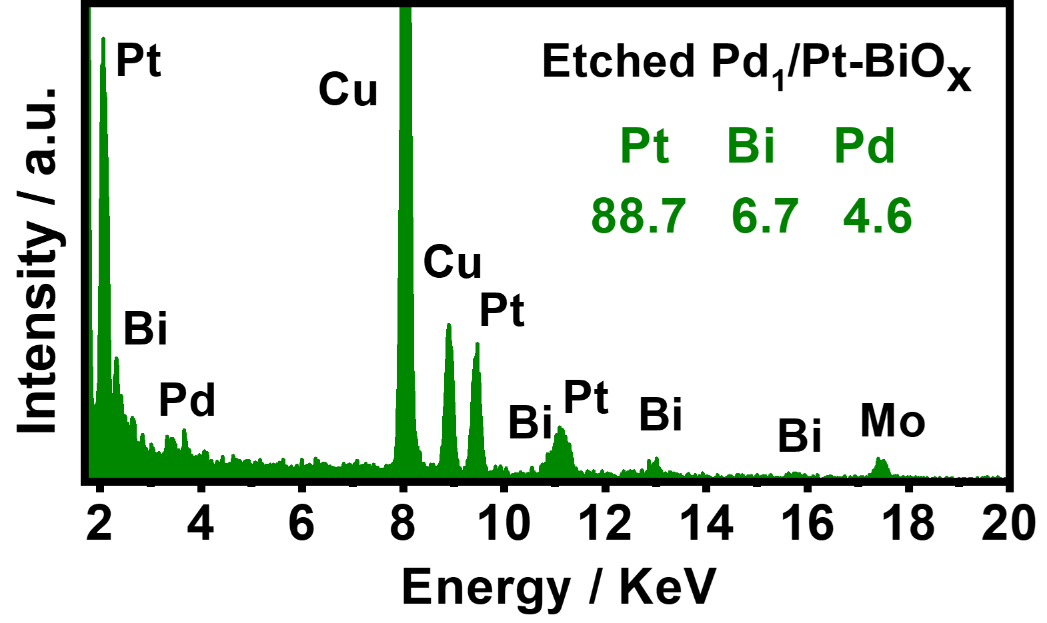


**Fig. S16** EDX spectrum of Pd_1_/Pt obtained by etching the BiO_x_ adatoms in Pd_1_/Pt-BiO_x_ via CV sweeping from -0.25 to 0.8 V vs. SCE for 400 cycles in Ar-saturated 0.5 M H_2_SO_4_ electrolyte at a scan rate of 100 mV s^-1^


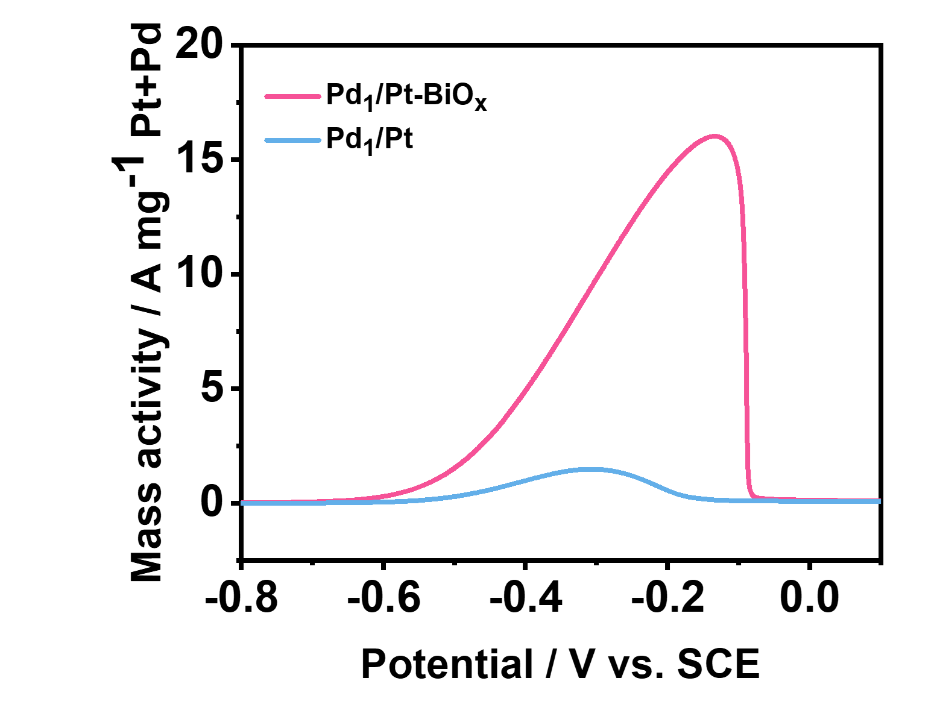


**Fig. S17** Positive-going CVs of Pd_1_/Pt-BiO_x_ before and after soaking in 0.5 M H_2_SO_4_ for 30 mins in Ar-saturated 1.0 M KOH + 1.0 M C_2_H_5_OH. Scan rate of 50 mV s^-1^


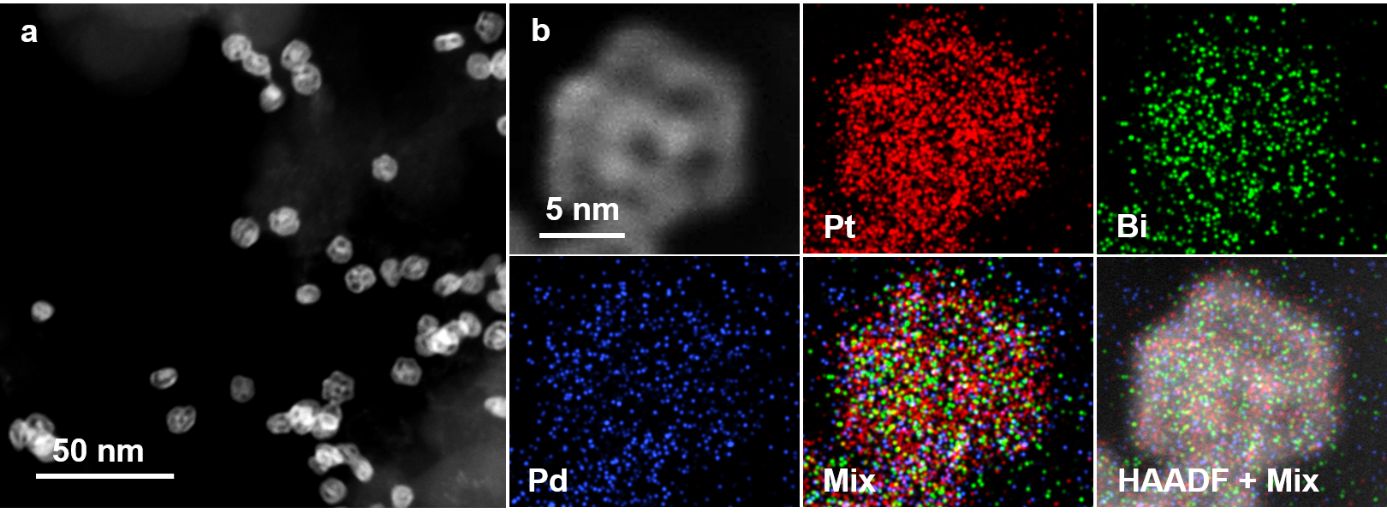


**Fig. S18** (**a**) HAADF-STEM image, and (**b**) EDX mapping images of Pd_1_/Pt. Red, green and blue signals indicate Pt, Bi and Pd, respectively


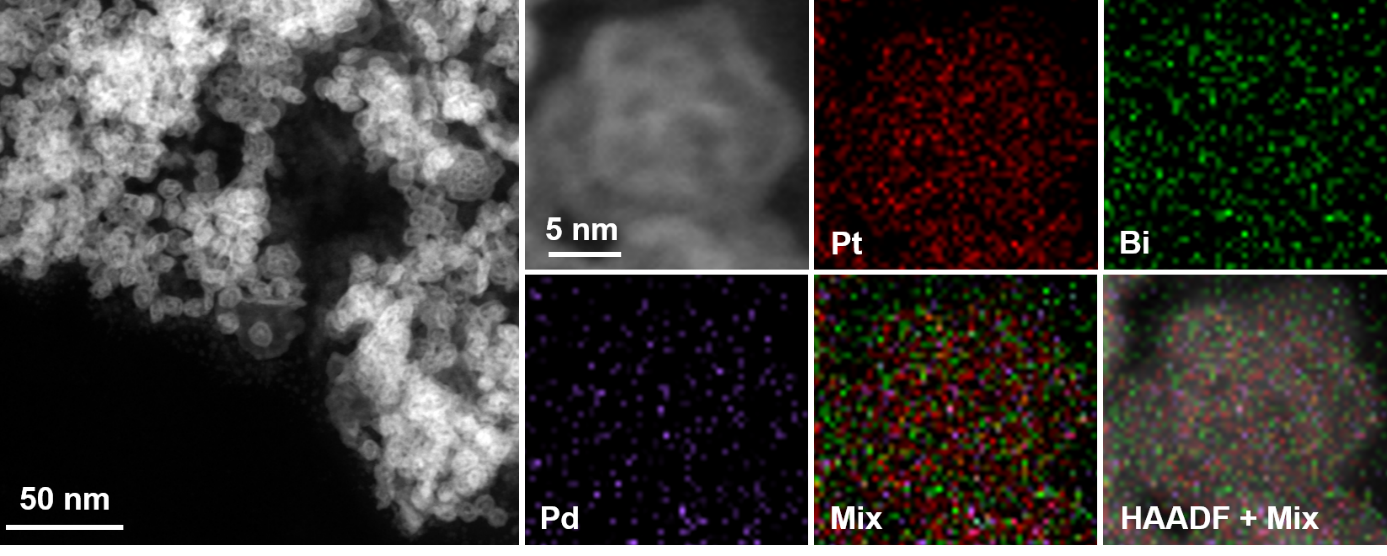


**Fig. S19** (**a**) HAADF-STEM image, and (**b**) EDX mapping images of Pd_1_/Pt-BiO_x_ after long-term CA operating at -0.4 V vs. SCE for 20000 s


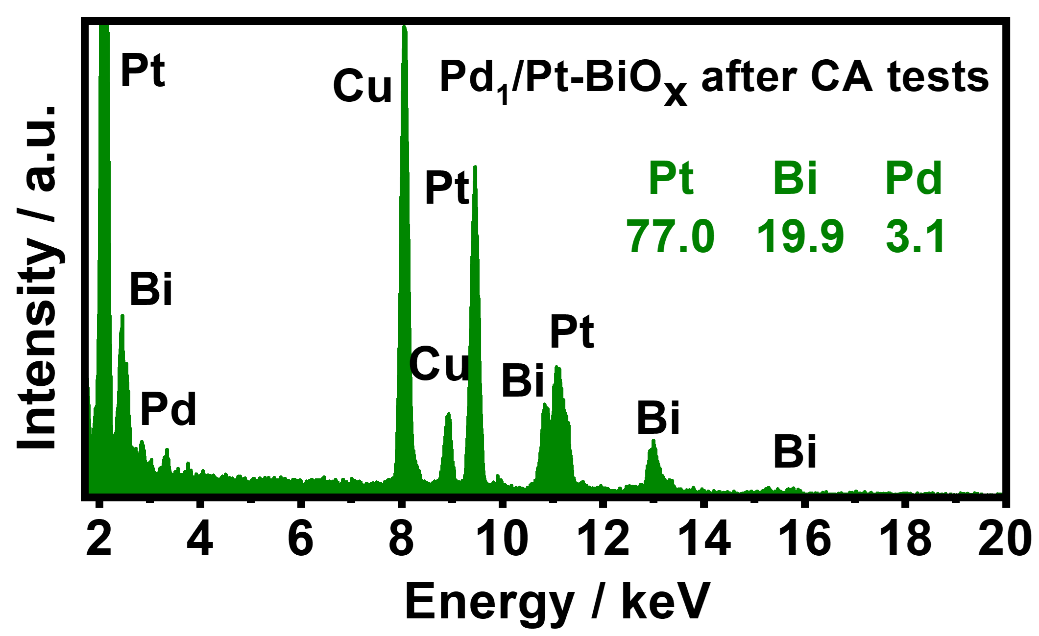


**Fig. S20** EDX spectrum of Pd_1_/Pt-BiO_x_ after long-term CA operating at -0.4 V vs. SCE for 20000 s

**
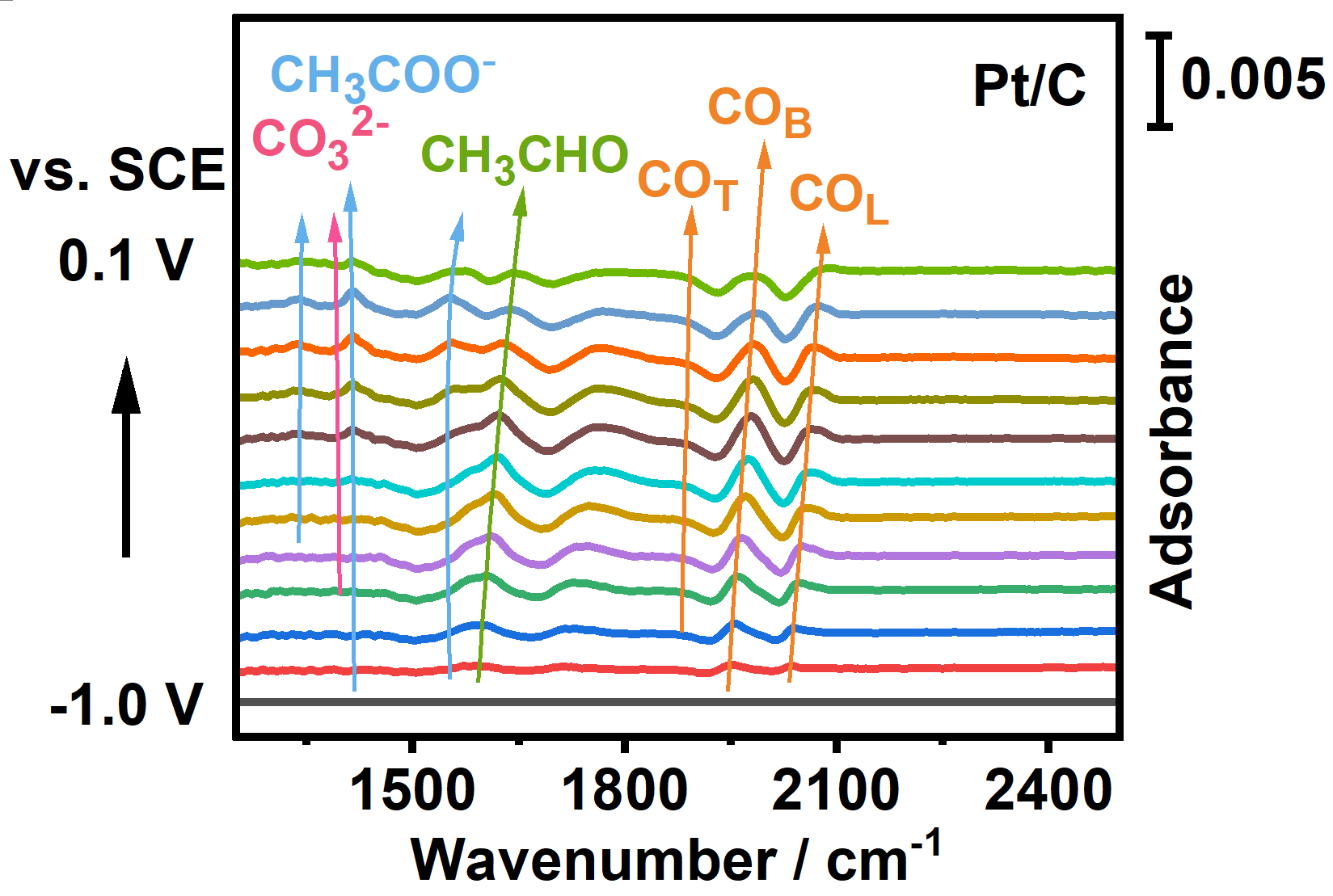
**

**Fig. S21** The in-situ FTIR spectra of Pt/C


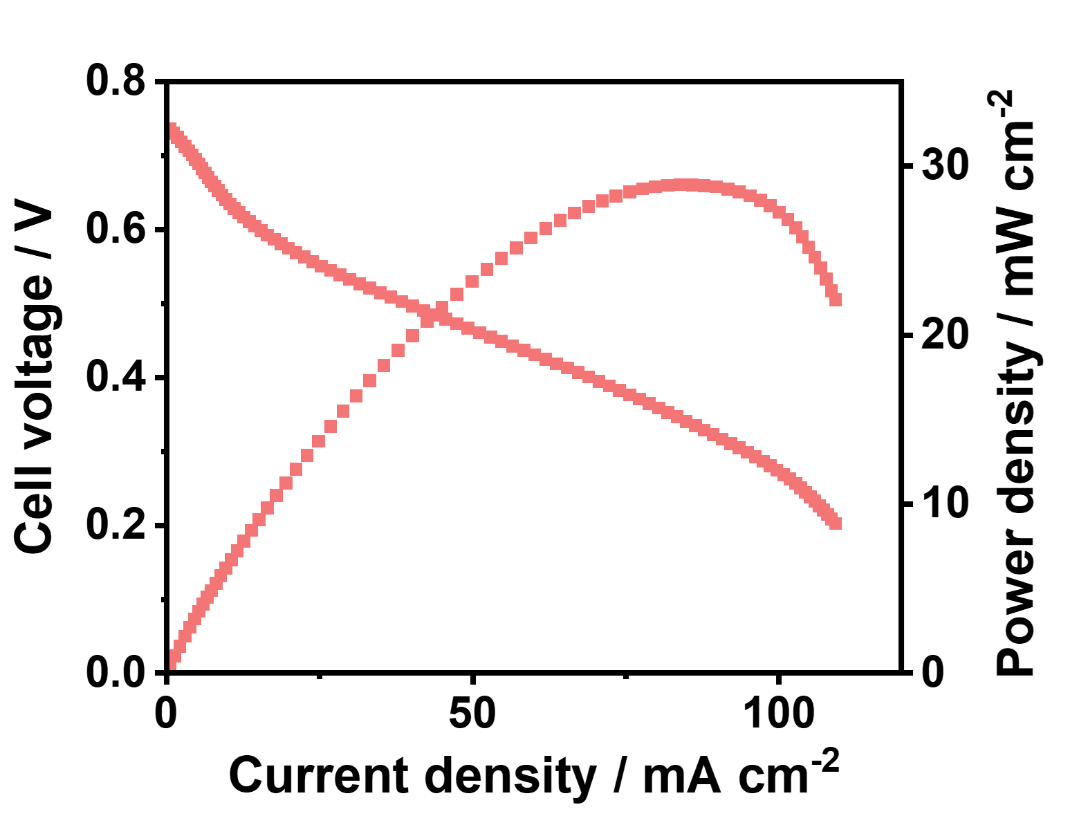


**Fig. S22** Power density curve of a DEFC assembled by using Pt/C as anodic electrocatalyst and commercial Pd/C as cathodic electrocatalyst


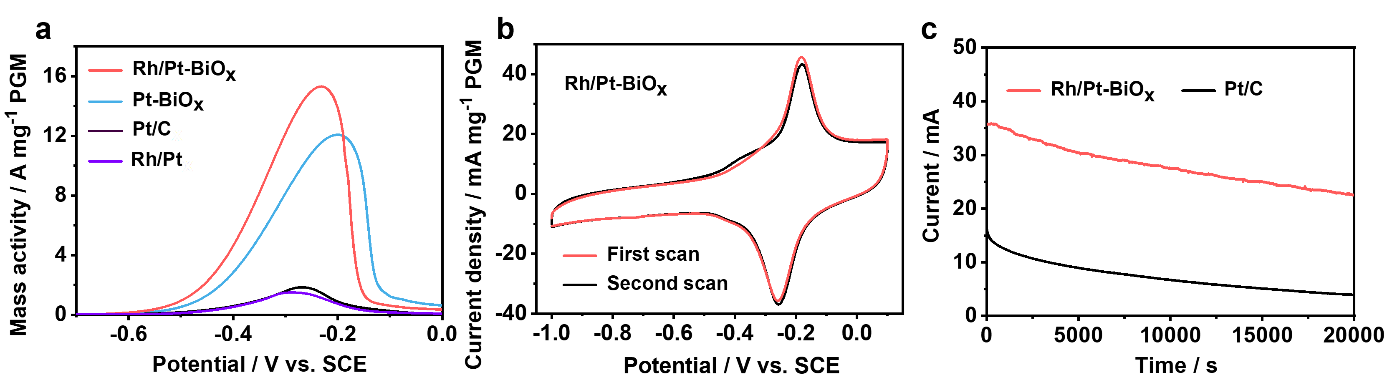


**Fig. S23** (**a**) Positive-going CVs of different electrocatalysts recorded in Ar-saturated 1.0 M KOH + 1.0 M CH_3_OH at a scan rate of 50 mV s^-1^. (**b**) CO-stripping curves of Rh/Pt-BiO_x_ in Ar-saturated 1.0 M KOH at a scan rate of 50 mV s^-1^. (**c**) CA curves of Rh/Pt-BiO_x_ and Pt/C electrocatalysts recorded at -0.4 V vs. SCE for 20000 s


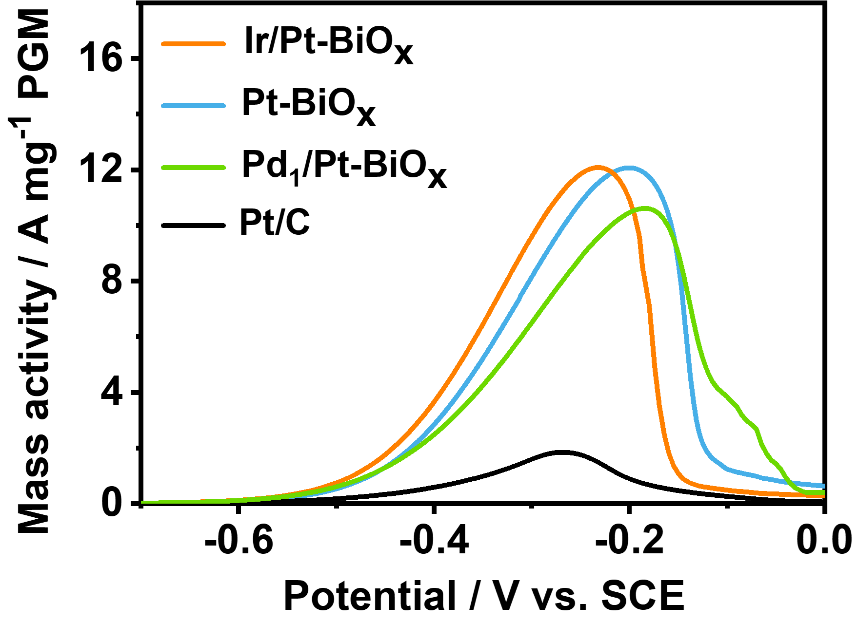


**Fig. S24** The positive-going MOR polarization curves of Ir/Pt-BiO_x_, Pt-BiO_x_, Pd_1_/Pt-BiO_x_, and Pt/C electrocatalysts recorded in Ar-saturated 1.0 M KOH + 1.0 M CH_3_OH at a scan rate of 50 mV s^-1^


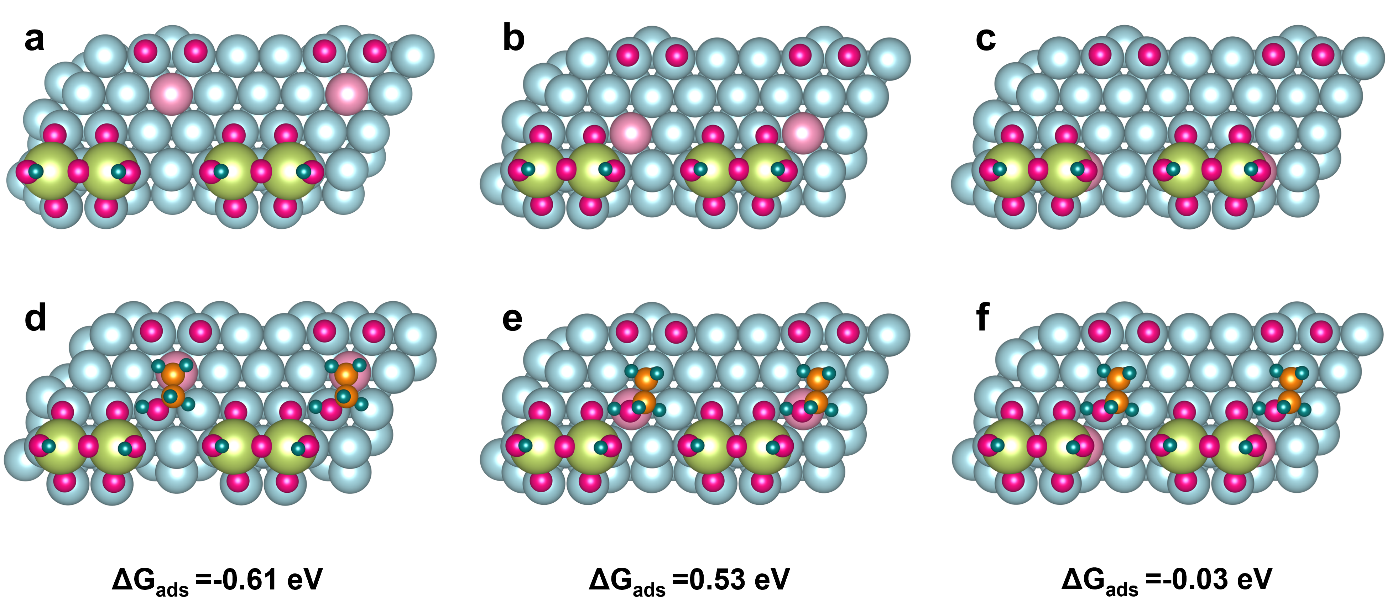


**Fig. S25** Top view for (**a-c**) structural models and (**d-f**) corresponding ethanol adsorption models of Pd_1_/Pt-BiO_x_ with different position of Pd_1_ in Pt-BiOx


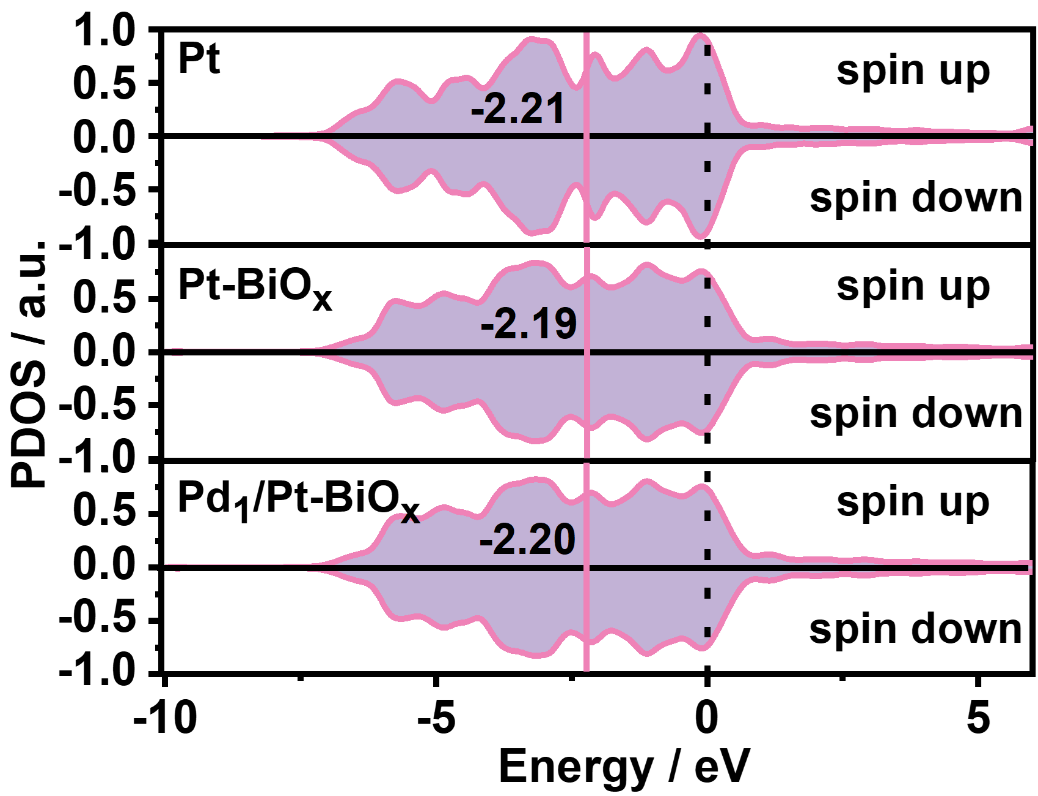


**Fig. S26** PDOS of surface Pt atom in Pt, Pt-BiO_x_, and Pd_1_/Pt-BiO_x_, respectively


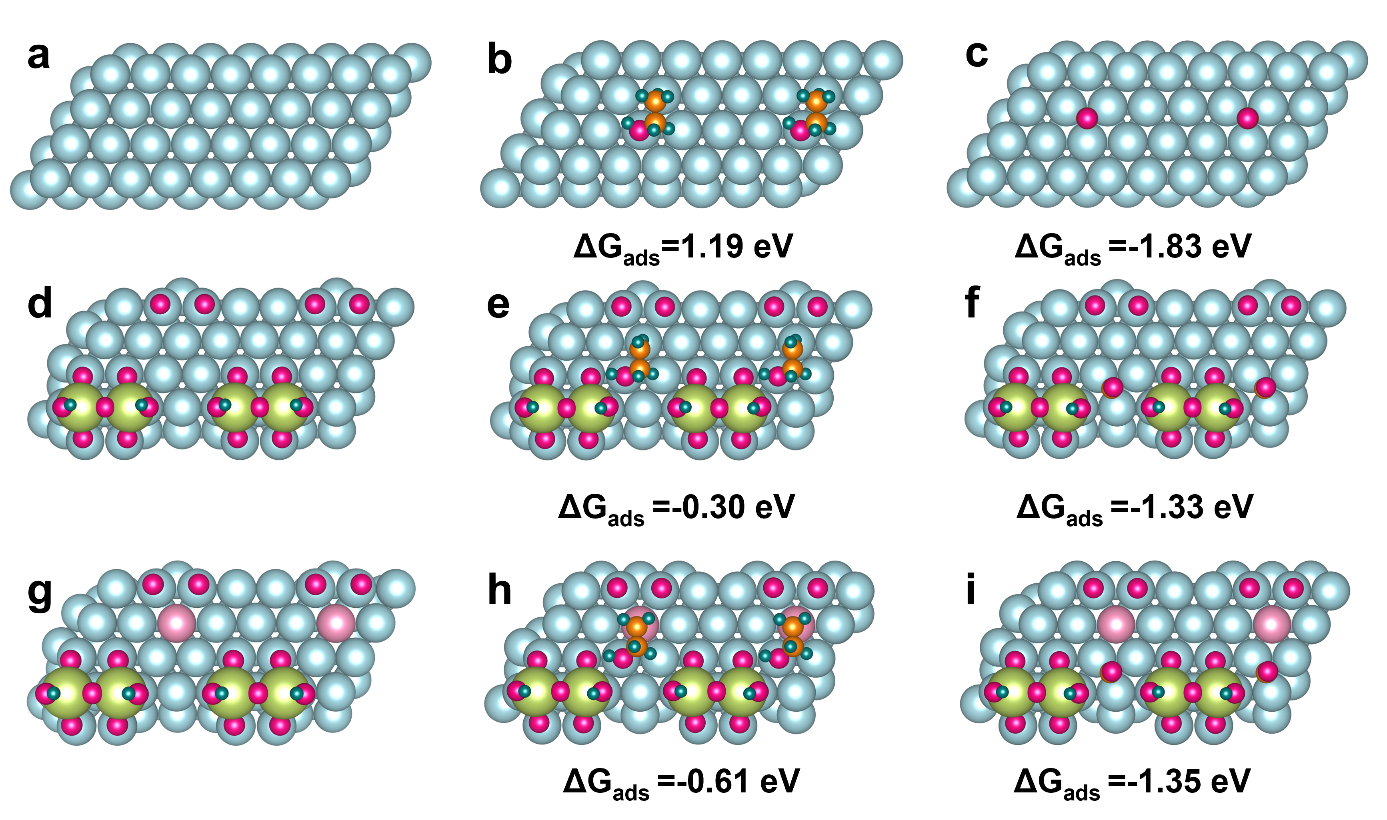


**Fig. S27** (**a-c**) Top view for Pt model, ethanol adsorbed Pt model, and CO adsorbed Pt model. (**d-f**) Top view for Pt-BiO_x_ model, ethanol adsorbed Pt-BiO_x_ model, and CO adsorbed Pt-BiO_x_ model. (**g-i**) Top view for Pd_1_/Pt-BiO_x_ model, ethanol adsorbed Pd_1_/Pt-BiO_x_ model, and CO adsorbed Pd_1_/Pt-BiO_x_ model. The Gibbs free adsorption energies are displayed below the corresponding models. Blue, herb green, orange, dark green, purple, and pink spheres represent Pt, Bi, C, H, O, and Pd atoms, respectively

**
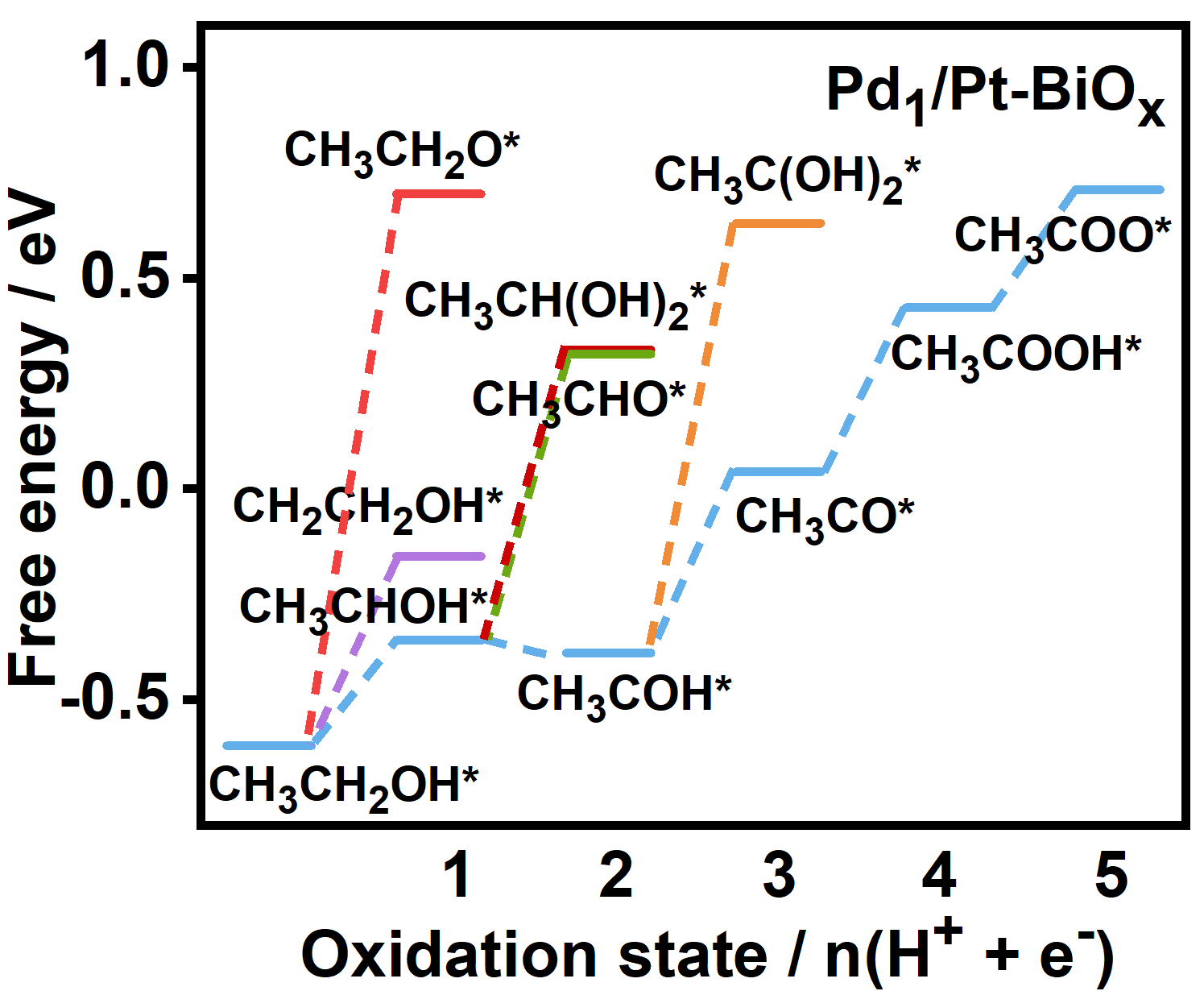
**

**Fig. S28** Potential energy diagram for EOR on Pd_1_/Pt-BiO_x_

**
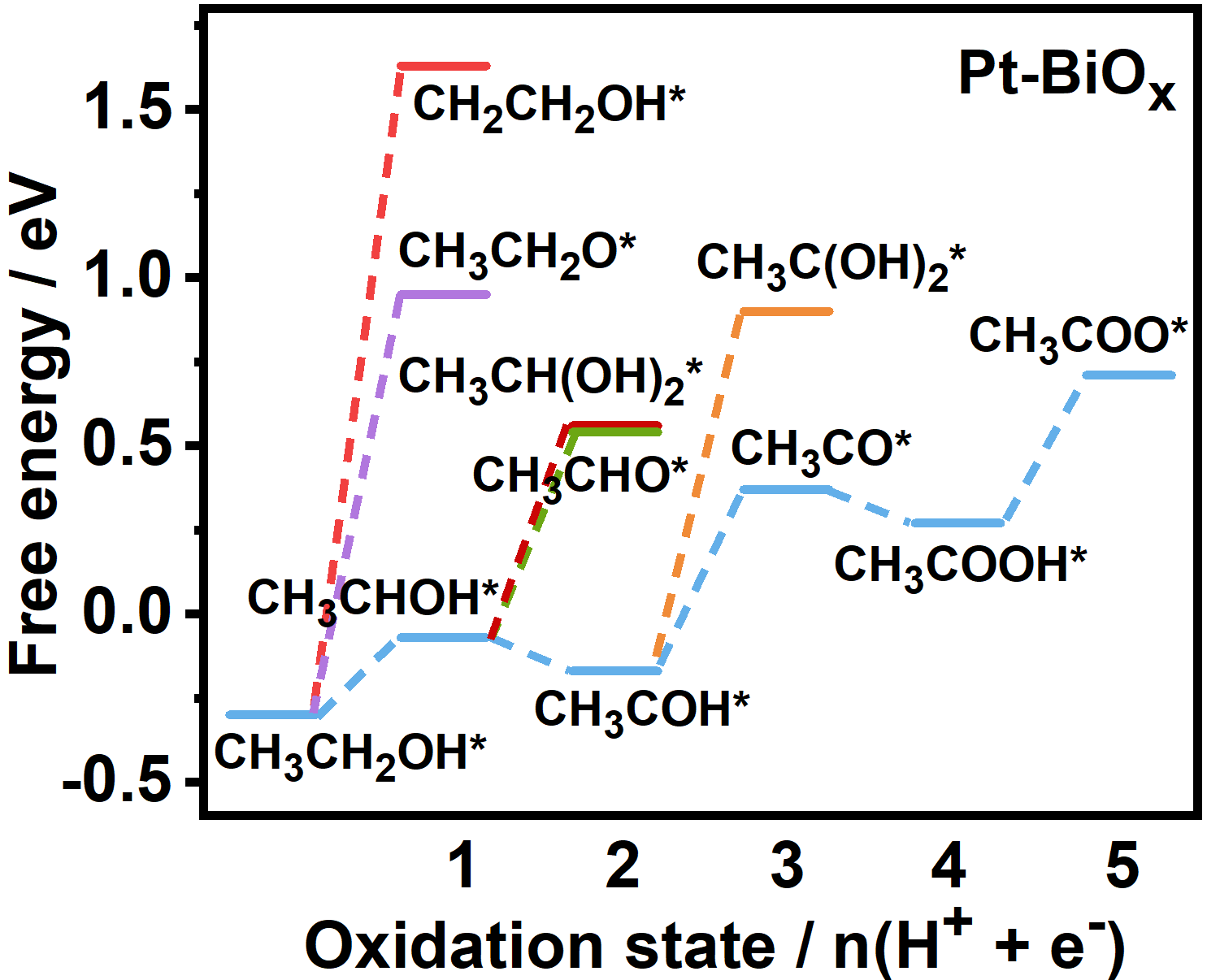
**

**Fig. S29** Potential energy diagram for EOR on Pt-BiO_x_

**Table S1** ICP results in different stages of Pd_1_/Pt-BiO_x_, Rh/Pt-BiO_x_, and Ir/Pt-BiO_x_


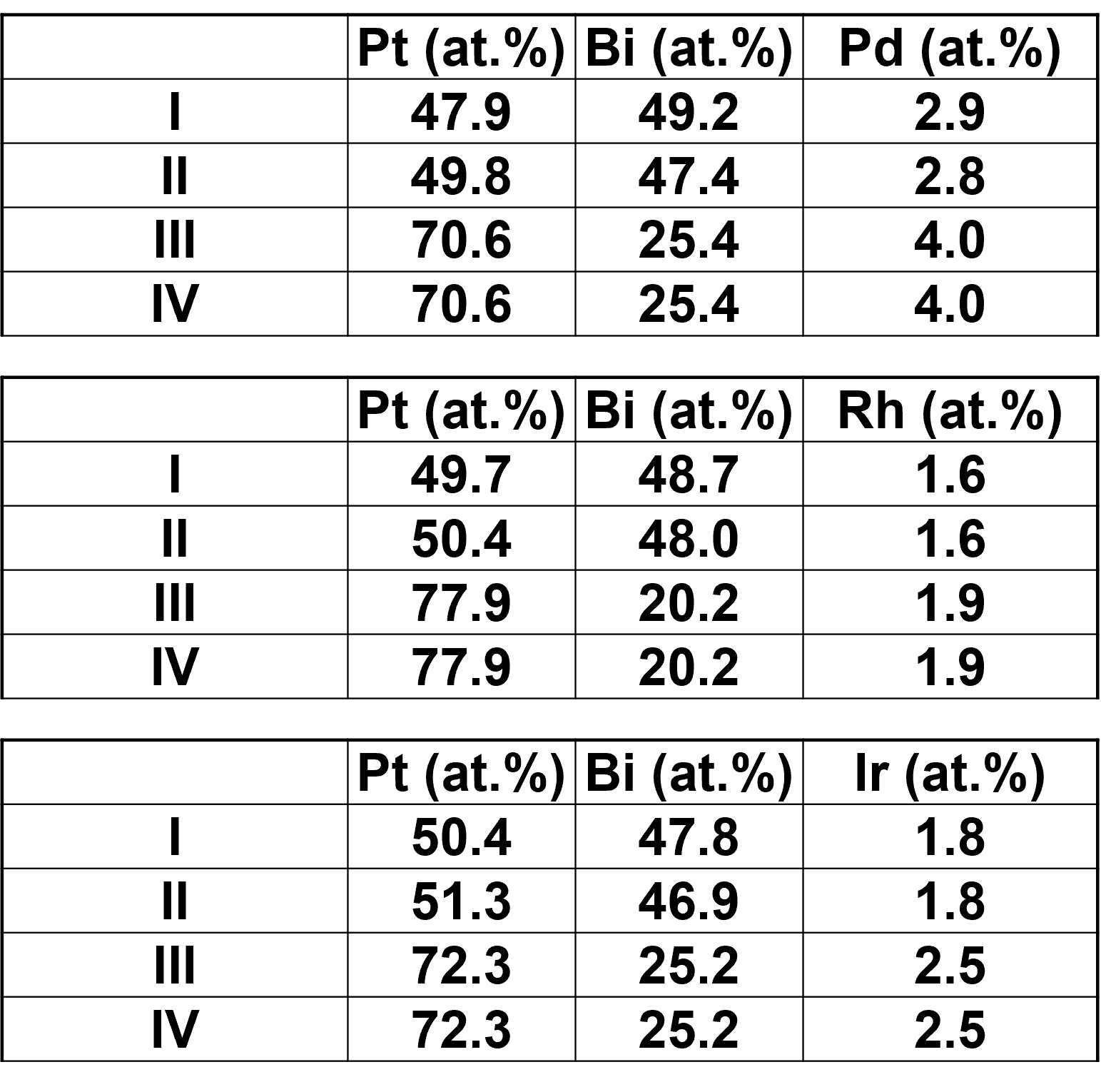


**Table S2** Comparison of the catalytic performances with the representative high performance EOR electrocatalysts in alkaline electrolytes that reported recently


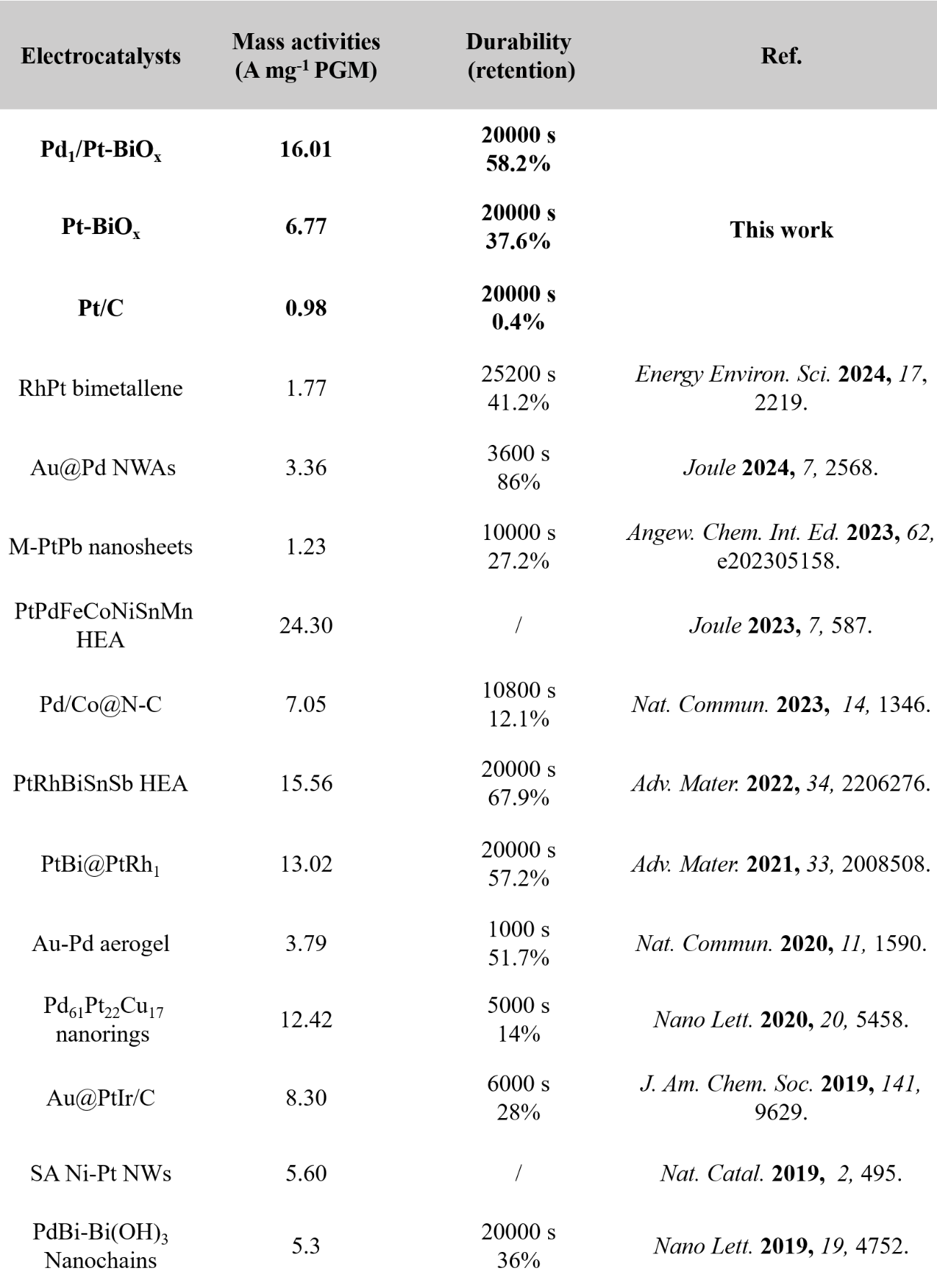

Supplement: Supplementary file 1 — Supplementary file1 (DOCX 14451 KB) [file 40820_2025_1678_MOESM1_ESM.docx]
